# Modulation of TLR/NF-κB/NLRP Signaling by Bioactive Phytocompounds: A Promising Strategy to Augment Cancer Chemotherapy and Immunotherapy

**DOI:** 10.3389/fonc.2022.834072

**Published:** 2022-03-01

**Authors:** Sajad Fakhri, Seyed Zachariah Moradi, Akram Yarmohammadi, Fatemeh Narimani, Carly E. Wallace, Anupam Bishayee

**Affiliations:** ^1^ Pharmaceutical Sciences Research Center, Health Institute, Kermanshah University of Medical Sciences, Kermanshah, Iran; ^2^ Medical Biology Research Center, Health Technology Institute, Kermanshah University of Medical Sciences, Kermanshah, Iran; ^3^ Student Research Committee, Kermanshah University of Medical Sciences, Kermanshah, Iran; ^4^ College of Osteopathic Medicine, Lake Erie College of Osteopathic Medicine, Bradenton, FL, United States

**Keywords:** TLR - toll-like receptor, NF-κB – nuclear factor-kappa B, NLRP, phytochemicals, chemotherapy, immunotherapy, signaling pathways, molecular pharmacology

## Abstract

**Background:**

Tumors often progress to a more aggressive phenotype to resist drugs. Multiple dysregulated pathways are behind this tumor behavior which is known as cancer chemoresistance. Thus, there is an emerging need to discover pivotal signaling pathways involved in the resistance to chemotherapeutic agents and cancer immunotherapy. Reports indicate the critical role of the toll-like receptor (TLR)/nuclear factor-κB (NF-κB)/Nod-like receptor pyrin domain-containing (NLRP) pathway in cancer initiation, progression, and development. Therefore, targeting TLR/NF-κB/NLRP signaling is a promising strategy to augment cancer chemotherapy and immunotherapy and to combat chemoresistance. Considering the potential of phytochemicals in the regulation of multiple dysregulated pathways during cancer initiation, promotion, and progression, such compounds could be suitable candidates against cancer chemoresistance.

**Objectives:**

This is the first comprehensive and systematic review regarding the role of phytochemicals in the mitigation of chemoresistance by regulating the TLR/NF-κB/NLRP signaling pathway in chemotherapy and immunotherapy.

**Methods:**

A comprehensive and systematic review was designed based on Web of Science, PubMed, Scopus, and Cochrane electronic databases. The Preferred Reporting Items for Systematic Reviews and Meta-Analyses guidelines were followed to include papers on TLR/NF-κB/NLRP and chemotherapy/immunotherapy/chemoresistance by phytochemicals.

**Results:**

Phytochemicals are promising multi-targeting candidates against the TLR/NF-κB/NLRP signaling pathway and interconnected mediators. Employing phenolic compounds, alkaloids, terpenoids, and sulfur compounds could be a promising strategy for managing cancer chemoresistance through the modulation of the TLR/NF-κB/NLRP signaling pathway. Novel delivery systems of phytochemicals in cancer chemotherapy/immunotherapy are also highlighted.

**Conclusion:**

Targeting TLR/NF-κB/NLRP signaling with bioactive phytocompounds reverses chemoresistance and improves the outcome for chemotherapy and immunotherapy in both preclinical and clinical stages.

## Introduction

Chemoresistance occurs when tumors mutate in response to cancer chemotherapy, yielding a more aggressive phenotype that results in chemotherapy failure (1). This has been a major obstacle in cancer chemotherapy and immunotherapy. Despite various attempts to overcome drug resistance and restore the sensitivity of chemotherapeutic drugs, the results thus far have been unsatisfactory (2). Several pathophysiological mechanisms and multiple dysregulated pathways are responsible for chemotherapy and immunotherapy resistance. Thus, revealing the critical dysregulated pathways in cancer chemoresistance would improve clinical outcomes and prevent/manage the development of chemoresistance, therefore limiting the progression and invasion of cancer (3). Amongst the dysregulated mediators, toll-like receptor (TLR) (4), nuclear factor-κB (NF-κB), and Nod-like receptor pyrin domain-containing (NLRP) (5), as well as the associated TLR/NF-κB/NLRP pathway, have been shown to contribute to cancer chemoresistance. In recent years, researchers have been seeking novel alternative agents with multiple targets, higher efficacy, and less side effects that can combat cancer chemoresistance.

Plant secondary metabolites are multi-targeting anticancer agents that target the cancer-associated pathways, including cellular senescence (6), Hippo signaling (7), Wnt/β-catenin (8), Janus kinase (JAK)/signal transducer and activator of transcription (STAT) (9), phosphoinositide 3-kinases (PI3K)/Akt/mammalian target of rapamycin (mTOR) (10), hypoxia-inducible factor-1α (HIF-1α) (11), and activator protein 1 (AP-1) (12). Phenolic compounds, alkaloids, terpenes/terpenoids, and sulfur-containing compounds demonstrated anticancer potential by modulating tumorigenic signaling pathways (6, 13). Emerging evidence has shown the influence of chemoresistance on cancer therapy (5, 14, 15). Although phytochemicals have exhibited critical regulatory roles in the modulation of TLR, NF-κB, and NLRP in combating cancer (16–18), there is no review report on the potential of phytochemicals in targeting TLR/NF-κB/NLRP pathway and pivotally interconnected pathways during chemoresistance. Thus, there is an imperative need to discover the precise dysregulated pathways involved in chemoresistance as well as to develop new strategies and alternative therapies to combat cancer chemoresistance. This is the first systematic and comprehensive review regarding crucial chemoresistance mechanisms and the therapeutic potential of plant secondary metabolites in combating cancer chemoresistance by targeting the TLR/NF-κB pathway and interconnected mediators. The need to develop novel phytocompound delivery systems to fight cancer chemoresistance is also highlighted.

## Resistance Mechanisms in Cancer Chemotherapy

Chemoresistance is one of the critical obstacles that affects the efficacy of anticancer drugs. Several factors contribute to the development of cancer chemoresistance. The most common cause of resistance to anticancer agents is the overexpression of energy-dependent transporters that export anticancer agents from the cancer cells (1). Consequently, decreased drug accumulation is another manner of chemotherapeutic resistance, which prevents drug-induced DNA damage and cancer cell apoptosis. Reduced sensitivity to drug-associated apoptosis plays a vital role in the resistance to anticancer drugs (19). Chemotherapy failure can be contributed in part to specific genetic and epigenetic alterations in addition to host factors. Cancer cells have a variety of genetic alterations depending on the tissue and patterns of oncogene activation and tumor suppressor gene inactivation. The use of powerful anticancer agents on cancer cells with these genetic factors leads to the development of drug-resistance mechanisms and the rapid achievement of chemoresistance in various cancer types. Host elements, such as rapid metabolism, poor absorption, and drug excretion, cause low serum levels of the drugs and thus a disposition towards chemoresistance. Host factors also reduce drug delivery to the tumor site, especially in solid bulky tumors with low cell penetration and high molecular weight. Additional mechanisms of cancer cell chemoresistance include receptor loss and mutations in the drug binding site (20).

The administration of various drugs has shown promising effects on chemotherapy with high cure rates by targeting multiple mechanisms of cell entrance. However, cancer cells may develop adaptations to resist these chemotherapeutic drugs, termed multidrug resistance (MDR). MDR strategies include reduced drug accumulation within the cancer cells, decreased uptake, increased efflux, and changes in the membrane lipid properties. These MDR mechanisms limit the apoptosis in cancer cells that are typically induced by anticancer agents, reduce DNA repair mechanisms, and dysregulate the cell cycle and checkpoints in cancer cells. An alternative method to overcome MDR is the use of combination therapy (21). Multidrug resistance proteins (MRPs) decrease the efficacy of anticancer drugs and decrease drug penetration in cancer cells. An additional therapeutic approach involves identifying MDR biomarkers before or during the treatment program (22, 23).

Reduced drug uptake and cell surface molecule mutations are other drug resistance mechanisms. Methods of drug entry into cells includes endocytosis and receptor binding followed by internalization of the drug. An example of the later strategy includes immunotoxins. Cancer cells with defective endocytosis are resistant to immunotoxins and toxins (24, 25).

Drug efflux from cancer cells is accomplished through various pumps. For example, ATP-binding cassette (ABC) transporters [e.g., P-glycoprotein (P-gp)] play an essential role in drug-related clinical resistance. P-gp levels are increased in several cancers through typical molecular mechanisms and directly correlate with chemotherapy resistance (26). Thus, P-gp expressing cells develop in tumors following *in vivo* exposure to chemotherapeutic agents. In certain cancer types, elevated P-gp resulted in clinical relapse and decreased P-gp showed therapeutic potential. Accordingly, drugs transported by P-gp have a low chemotherapy response. Therefore, P-gp inhibitors have therapeutic potential in chemotherapy. Despite the high expression of P-gp in solid tumors (e.g., colon and renal cancer), no chemoresistance was shown, implying other methods of drug resistance are at work. Recent research has demonstrated the involvement of the ABC transporter multixenobiotic resistance (MXR) and MRPs in drug resistance. These results express the need to consider treatment with nonspecific inhibitors of ABC transporters or a cocktail of specific inhibitors with the broadest spectrum effect (27).

**Figure 1:**
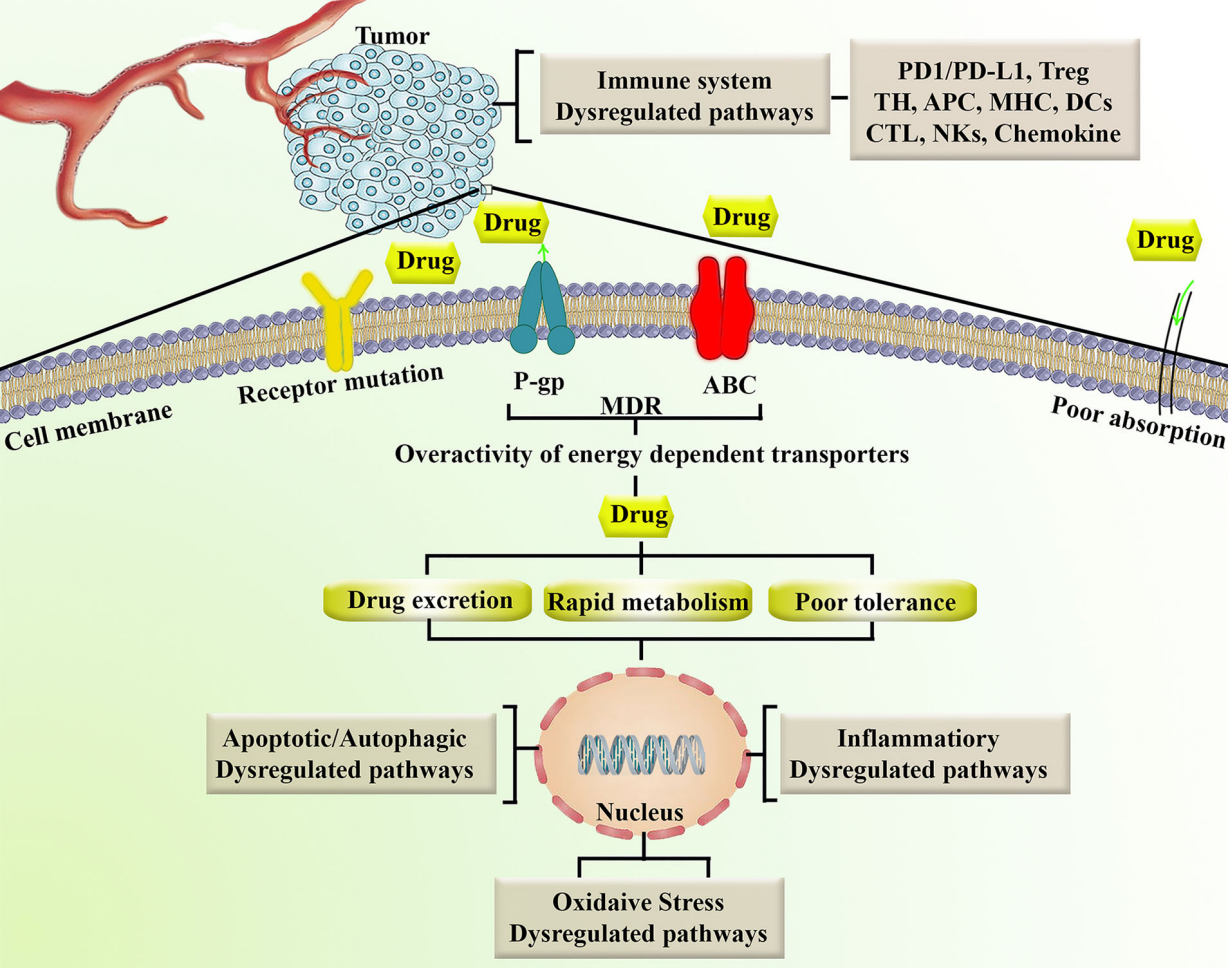
Representation of major factors involved in chemoresistance. ABC, ATP-binding cassette; APC, antigen-presenting cell; CTL, cytotoxic T lymphocytes; DCs, Dendritic cells; MDR, multidrug resistance; MHC, major histocompatibility complex; NKs, natural killer cells; P-gp, P-glycoproyein; PD1/PD-L1, programmed cell death-1/programmed cell death-ligand 1; TH, T helper; Treg, T regulatory.

Another mechanism of chemoresistance is reduced intracellular drug activation and improved drug inactivation by phase I and/or II enzymes in the intestine, liver, and tumor (28). Cytochrome P450s (CYPs) are critical phase I metabolism enzymes that act as an oxidation catalyzer in many anticancer drugs. Genetic mutations in CYPs have shown significant effects on the toxicity and efficacy of anticancer agents that are primarily metabolized by CYPs. Carboxylesterase, deoxycytidine, cytidine deaminase, kinase, and epoxide hydrolase are enzymes involved in the detoxification and/or activation of some anticancer drugs. Mutations in these enzymes may alter their activity and play a role in the chemoresistance process. Cancer cells can become drug-resistant by decreasing drug activation through the reduction or mutation of kinases (29). The aforementioned dysregulated mechanisms that contribute to chemoresistance lead to rapid metabolism/excretion, poor tolerance and downstream oxidative stress, apoptosis/autophagy, and inflammation. **Figure 1** summarizes the major factors involved in cancer chemoresistance.

In addition to the above mechanisms, evasion of apoptosis/autophagy/necrosis is critical to tumor resistance. In any pathological condition, programmed cell death pathways, including apoptosis, and autophagy are the cause of death through intracellular pathways. Such cell death programs may cooperatively determine the fate of malignant neoplasms. Programmed necrosis and apoptosis always contribute to cell death, however, autophagy can play either pro-death or pro-survival roles (30). The mitochondrial (intrinsic) pathway and death receptor (extrinsic) pathway are two methods of apoptosis. Initiation of both pathways ultimately proceeds through caspase-related cascades. A group of cysteine proteases plays an important role in inflammation and apoptosis by cleaving a variety of nuclear and cytoplasmic mediators. Apoptotic caspases can be either initiator or executioner caspases. These include initiator caspase-2, caspase-8, caspase-9, and caspase-10 and executioner caspase-3, caspase-6, and caspase-7. In regard to the initiator caspases, CD95 (APO-1/Fas) and tumor necrosis factor (TNF)-related apoptosis-inducing ligand (TRAIL) are members of the TNF receptor superfamily of death receptors which recruits caspase-8, forming a multimeric complex at the plasma membrane that subsequently activates caspase-3. Caspase-8 causes the release of cytochrome c by increasing the permeability of the outer mitochondrial membrane through cleavage of Bid, a BH3-only protein, and translocation to the mitochondria (31, 32). Mitochondrial apoptosis-induced channel (MAC) also increases the release of cytochrome c, which activates caspase-3 through the creation of pro-caspase-9, apoptotic protease activating factor 1 (Apaf-1), and the apoptosome. The apoptosome converts pro-caspase-9 to its active form, caspase-9, which then activates caspase-3 (31). Apaf-1 forms an oligomeric apoptosome which determines the apoptotic pathway upon binding with ATP and cytochrome c. Such apoptotic pathways are controlled by various negative and positive regulators, which play a critical role in chemoresistance (33).

The B-cell lymphoma 2 (Bcl-2) family of proteins consists of anti-apoptotic and pro-apoptotic proteins. The former includes B-cell lymphoma-extra-large (Bcl-xL), Bcl-2, and myeloid-cell leukemia 1 (Mcl-1), while the latter includes Bcl-2-associated X protein (Bax), Bcl-2 homologous antagonist/killer (Bak), and BH3. Bak and Bax enhance the formation of MAC, while Mcl-1, Bcl-2, and Bcl-xL inhibit its formation (34). Apart from the established roles of Bak and Bax in apoptosis, the ratio of pro-apoptotic/anti-apoptotic proteins is what governs apoptosis, rather than individual protein expression. In cancer cells, the upregulation of anti-apoptotic mediators allows cells to evade apoptosis. This also allows cancer cells to escape apoptosis even when exposed to chemotherapeutic drugs, which would otherwise induce apoptosis in susceptible cells (35). Thus, dysregulation of the pro-apoptotic/anti-apoptotic protein ratio is another mechanism of chemotherapy resistance due to decreased apoptosis of cancer cells.

In cancer cells, the activities of both pro- and anti-apoptotic mediators are controlled by Jun amino terminal kinase (JNK) and p38-mitogen-activated protein kinase (MAPK). The latter increases p53 and apoptosis in chemoresistant cells through the protein kinase B (Akt)/Forkhead box O3 (FoxO) pathway. Insulin-like growth factor 1 suppresses apoptosis *via* casein kinase 2 and PI3K/Akt pathways, which in turn arrests Smac/DIABLO release and suppresses caspase activity (36). In addition, p53 mutations reduce chemoresistance through modulatory roles on mitochondrial function, leading to increased chemosensitivity. The overexpression of tissue inhibitor of metalloproteinase-1 (TIMP) compromises chemotherapy response *via* NF-κB and PI3K/Akt signaling pathways (37). The actin-bundling protein, fascin, plays an important role in breast cancer chemoresistance by increasing the level of anti-apoptotic proteins and blocking the entry of the pro-apoptotic proteins caspase-3 and caspase-9 (37). Notch-1 and survivin are other signaling pathways that contribute to chemoresistance by activating targets involved in cell survival, thereby inhibiting apoptosis in tumor cells (38).

Other mechanisms of tumor resistance are related to dysregulated autophagy. Autophagy allows cells to regain ATP and vital biosynthetic factors in tumor microenvironments that are hypoxic and starved, promoting cancer cell survival. Autophagy acts as a critical modulator of intracellular hemostasis, tumor suppression, aging, cell death, and tumor chemoresistance (39). However, autophagy acts as a double-edged sword, as it also plays a role in the initiation, growth, development, and invasion of tumor cells. Accordingly, the PI3K/Akt/mTOR signaling pathway plays a crucial role in autophagy by modulating cell growth, cell survival, protein synthesis, motility, cell metabolism, cell death, and chemoresistance (39). This also downregulates the pro-apoptotic mediators Bim and Bad (40). Apoptotic mediators and autophagy are also regulated by upstream JNK and p38MAPK, thereby playing a vital role in modulating chemoresistance (41). Many anticancer drugs disrupt the balance between autophagy and apoptosis by altering the genetic/epigenetic phenotype and inhibiting PI3K/Akt/mTOR in cancer cells, thereby leading to the development of chemoresistance (41).

## Resistance Mechanisms in Cancer Immunotherapy

Immunotherapy resistance is a primary and/or acquired resistance of tumor cells to immunotherapy (42). The inhibitors of programmed cell death-1/programmed cell death-ligand 1 (PD-1/PD-L1) increase the release of interferon γ (IFN-γ) and upregulate the JAK/STAT signaling pathway. This activates IFN regulatory factor 8 (IRF8), causing hyperprogression (HPD) (43). HPD is a primary form of drug resistance (44) associated with mutations of epidermal growth factor receptor (EGFR) [33], murine double minute (MDM) gene (43), and chromosome 11 region 13 (43).

Attenuation of immune checkpoints potentially stimulates regulatory T cells (Tregs), creating an immunosuppressive microenvironment and modulating autoantigenicity antigen shedding or endocytic antigens to mediate immune escape (45). Such conditions trigger the polarization of immunosuppressive cells, such as M2 macrophages, antigen-presenting cells (APCs), and myelocytes, producing immunosuppressive cytokines. This also stimulates T helper type 1 (TH1) and TH17-mediated inflammatory conditions to upregulate oncogenic pathways and accelerate tumor growth and immunotherapy resistance (43, 46). Consequently, dendritic cells (DCs), B lymphocytes, monocyte-macrophages, and other APCs such as fibroblasts, endothelial cells, mesothelial cells, and epithelial cells are interconnected with tumor-specific antigen (TSA)/tumor-associated (TAA), conferring immunogenicity and T cell infiltration in tumors. Autophagy and the endoplasmic reticulum (ER) determine the tumor-associated immunogenicity of cell death (47). Dysregulation of antigen presenting signaling pathways, including mutations of the proteasome, transporters, and major histocompatibility complex (MHC), is cross-talked with T cell activity and tumor immune escape. MHC mutations are classified into structural defects, changes in the receptor-binding domain, and epigenetic changes (48). In some cancer types, tumor cells are able to escape lysis mediated by cytotoxic T lymphocytes (CTLs) and natural killer cells (NKs) through the overexpression of MHC-I. This allows tumor cells to escape the immune system (49).

The dysregulation of emerging signaling pathways in tumor cells is another mechanism of immunotherapy resistance. For instance, IFN-γ, produced by T cells and APCs, binds to related receptors to activate JAK2 (50). This leads to interaction with STAT1, which modulates downstream cascades. IFN-γ allows tumor cells to escape the immune system by increasing the expression of PD-L1 on the surface of tumor cells (51). IFN-γ also upregulates C-X-C motif chemokine ligand (CXCL)-9 and CXCL-10 chemokines and promotes antitumor immune cell effects (51). Additionally, IFN-γ exerts pro-apoptotic and antitumor properties through binding to cell surface receptors and triggering downstream mediators to suppress tumor cells (51). In patients receiving immunotherapeutic agents, tumor cells alter IFN-γ and JAK/STAT1 signaling pathways. Tumor analysis of chemoresistant patients receiving anti-cytotoxic T-lymphocyte-associated protein 4 (CTLA-4) agents had mutations in IFN-γ pathway genes, JAK1/2, and interferon regulatory factors (52). This allowed tumor cells to evade T cells, thus resisting the anti-CTLA-4 treatment. Loss of polybromo and BRG1-associated factors (PBAF) complex increased the ability of chromatin to regulate IFN-γ as well as increased production of CXCL-9/CXCL-10 to recruit T cells to tumor tissue (53). In human cancers, expression of Pbrm1 and Arid2 is correlated with the presentation of T cell cytotoxicity genes, leading to immunotherapy resistance (53).

Spranger et al. (54) demonstrated that the infiltration of T cells and recruitment of DCs into the total mesorectal excision (TME) could be suppressed by tumor-intrinsic β-catenin activation *via* decreased expression of CCL4. Because DCs prevent migration into epithelial-to-mesenchymal transition (EMT), no antigen can be presented to T cells, halting their cytotoxic effects. From another mechanistic point, upregulation of MAPK signaling damages the function and infiltration of tumor-infiltrating lymphocytes through the expression of vascular endothelial growth factor (VEGF) and cytokines such as interleukin-8 (IL-8) (55). Under these conditions, induction of Tregs ultimately leads to tumor immune evasion. Loss of tumor suppressor phosphatase and tensin homolog (PTEN) leads to activation of PI3K signaling, which is associated with increased anti-inflammatory cytokines, such as VEGF and C–C motif chemokine ligand 2 (CCL2), reduced infiltration of CD8+ T cells into tumors, and decreased IFN-γ expression, conferring resistance of PD-1 blockade therapy against tumors (56).

Tumor cells develop immunotherapy resistance by altering tumor cell metabolism through multiple metabolic changes, termed tumor metabolic reprogramming (57). One such mechanism utilizes aerobic glycolysis to create a hypoxic acidic environment which prevents normal metabolism of immune cells and impairs T cell function and infiltration (58). Furthermore, glucose consumed by tumor cells may restrict T cell metabolism, which leads to inhibition of mTOR, decreased glycolytic capacity in T cells, and production of intracellular IFN-γ (59).

## TLR/NF-κB/NLRP Signaling Pathway in Cancer Initiation and Progression and Chemoresistance

TLRs are members of the type I transmembrane proteins and are conserved pattern-recognition receptors (PRRs) that are activated by various pathogen-associated molecular patterns (PAMPs). These membrane proteins are heavily expressed on the surface of several cells, including monocytes, macrophages, and DCs. The three constructional domains of TLRs’ include a leucine-rich repeats (LRRs) motif, a transmembrane domain, and a cytoplasmic domain. Each of these domains have a specific function. For example, pathogen recognition is performed by the LRR motif, while signal initiation is performed by interaction of the TIR domain with the signal transduction adaptors. This receptor family is extremely important for pathogen recognition by the innate immune system (60, 61). Recently, several reports have indicated the association between cancer and TLRs. Specifically, the TLR4 signaling pathway is the most tightly linked with inflammatory response and cancer initiation and progression (16).

TLRs are involved in tumor progression, however they may display either anti- or pro-tumor metastasis and growth features (62). Activation of TLR4 increased IL-6 and IL-8 production in breast cancer (63). In some cancers, TLR4 induced the production of nitric oxide and IL-6 (64). In prostate cancer cells, TLR4 activation enhanced the expression of transforming growth factor-β1 (TGF-β1) and VEGF, which promoted tumor progression (65). Some studies have shown poorer outcomes for breast, colon, and pancreatic cancers when TLR4 is overexpressed (63, 64). The myeloid differentiation factor 88 (MyD88) pathway of TLR4 has been shown to improve carcinogenesis. Yusef et al. (66) found that TLR4 demonstrated antitumor activity in skin cancer. The role of TLR4 should be further evaluated in various cancer types. Overall, these results suggest that the release of various inflammatory mediators, cytokines, and chemokines activates TLR4 and may participate in cancer formation.

TLRs are strong actuators of the inflammatory response, activation of which triggers the production of interferons, chemokines, cytokines, and NF-κB. The NF-κB pathway plays a crucial role in various diseases through regulation of cell proliferation, differentiation, immunity, and apoptosis (67). The NF-κB family consists of five crucial parts: p50, p52, p65/RelA, c-Rel, and RelA. NF-κB acts as a transcription factor by binding DNA, which activates gene transcription. Several genes involved in the progression and development of cancer are regulated by NF-κB, such as those involved in proliferation, apoptosis, and migration. Improper or constitutive NF-κB activation has been found in many malignant human tumors (68).

Typically, NF-κB is bound to IκB (IκB) in the cytoplasm. In times of stress, reactive oxygen species (ROS) and inflammatory stimuli degrade the IkB complex to activate NF-κB, releasing inflammatory cytokines such as tumor necrosis factor-alpha (TNF-α), IL-1, IL-6, and IL-2. This inflammatory cascade suppresses apoptosis and induces cellular invasion, proliferation, and metastasis, aiding in chemoresistance [52]. Prevailing reports have shown that activation of NF-κB by tumors assists in the development of chemotherapy resistance. NF-κB activation plays a key role in hindering the effectiveness of chemotherapeutic agents. Tumor cells exposed to chemotherapeutic drugs or radiation showed increased activation of NF-κB, which enforced the expression of MDR P-gp. Meanwhile, NF-κB suppression improved the apoptotic response to radiation therapy (69).

Members of the NLR family play an essential role in the signaling pathways of the innate immune system by activating or inhibiting inflammasomes. Damage-associated molecular patterns (DAMPs) and PAMPs activate NLRs and absent in melanoma 2 (AIM-2)-like receptors (ALRs), which bind to associated cytosolic domains to activate caspases. As a result, caspases upregulate IL-18 and IL-1β, which results in apoptosis and pyroptosis (70). On the other hand, dysregulation of NLR contributes to various autoimmune and inflammatory diseases. Thus, NLR can play a role in tumor suppression or tumor promotion in the initiation, development, and regression of cancer (71). Therefore, targeting the TLR/NF-κB/NLRP signaling pathway may facilitate improvement in the regulation of cancer initiation/progression and associated chemoresistance.

## TLR/NF-κB/NLRP Signaling Pathway in Cancer Immunotherapy

**Figure 2:**
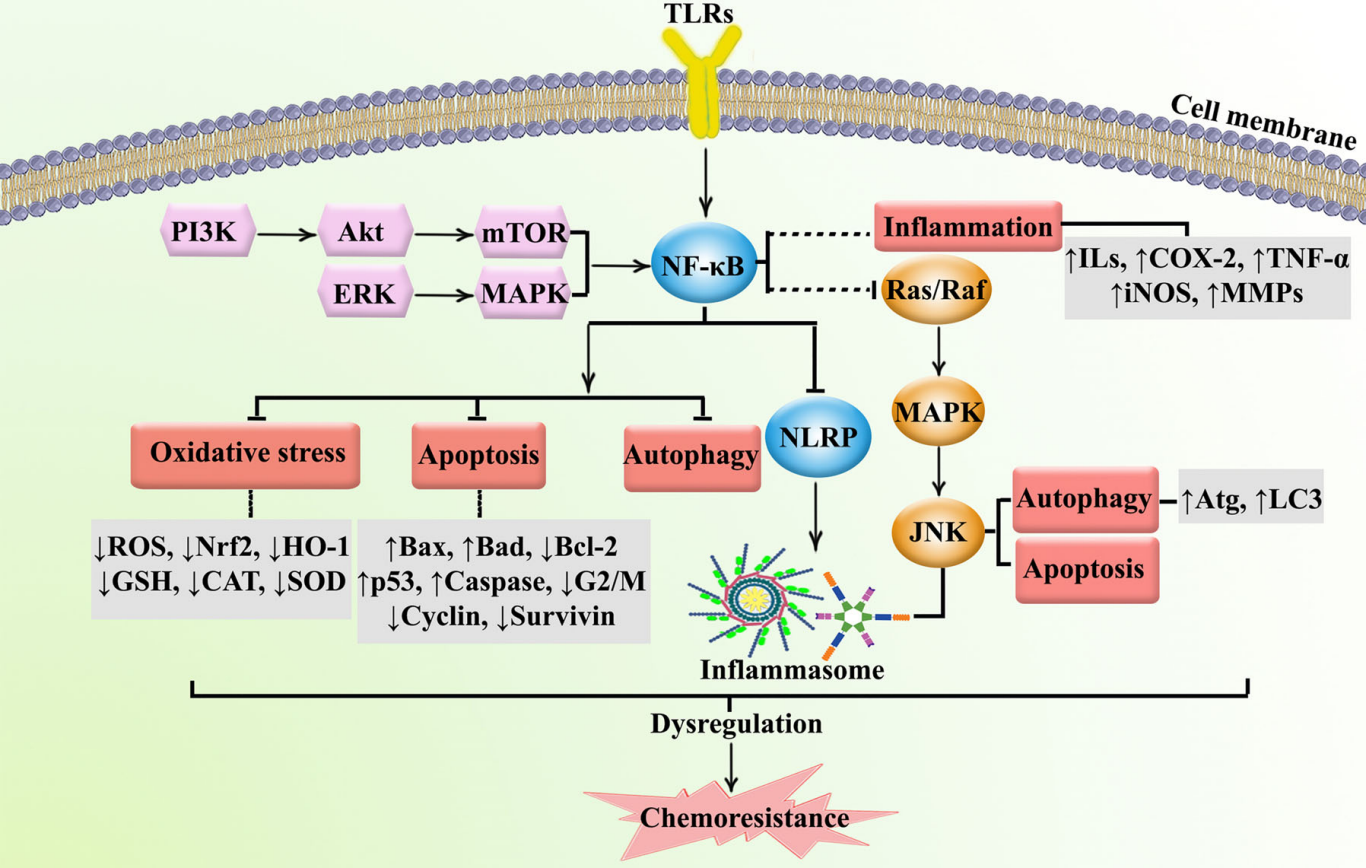
Major dysregulated pathways in cancer chemoresistance. Atg, autophagy-related; CAT, catalase; COX-2, cyclooxygenase; ERK, extracellular-regulated kinase; GSH, glutathione; HO-1, heme oxygenase 1; ILs, interleukins; iNOS, inducible nitric oxide synthase; JNK, c-Jun N-terminal kinase; LC3, microtubule-associated protein 1A/1B-light chain 3; MAPK, mitogen-activated protein kinase; MMP, matrix-metalloproteinase; mTOR, mammalian target of rapamycin; NLRP, nod-like receptor pyrin domain-containing; Nrf-2, nuclear factor-erythroid factor 2-related factor 2; PI3K, phosphoinositide 3-kinases; ROS, reactive oxygen species; SOD, superoxide dismutase; TNF-α, tumor necrosis factor-α.

TLRs are part of a family of recognition receptors which play a pivotal role in the host immune system (72–74). TLRs are expressed by B cells, macrophages, monocytes, NK cells, mast cells, neutrophils, and basophils. TLRs stimulate pro-inflammatory chemokines and cytokines to activate the innate and adaptive immune systems. The activation of TLR4 can induce associated adaptor proteins, including MyD88, TIR domain-containing adapter molecule 1 (TICAM1), TIR domain-containing adapter molecule 2 (TICAM2), and TIR domain-containing adaptor protein (TIRAP). Some ligands (e.g., lipopolysaccharides and toxins) bind TLRs to activate the immune response. According to Nagai et al. (75), the co-receptor myeloid differentiation factor-2 (MD-2) increased the translocation of TLR4 to form a heterotrimer of CD14/TLR4/MD-2 (76). This may lead to two distinct signaling pathways, the MyD88 pathway and the toll/IL-1R domain-containing adapter-inducing IFN-β (TRIF) pathway. Tumor necrosis factor receptor-associated factor 6 (TRAF6) activates extracellular signal-regulated kinase (ERK), MAPKs, and the p38 signaling pathway. Alternatively, TLR4 activates the MyD88-independent pathway to upregulate NF-κB and suppress IκB kinase epsilon (IKK). MyD88-dependent and MyD88-independent pathways also contribute to host defense and engage the immune response. In addition, TLRs activate IRFs, which increase the transcription of interferon-α (IFN-α) and interferon-β (IFN-β) (77). NLRP is the downstream mediator of NF-κB, which is interconnected with the inflammasomes. Inflammasomes are receptors/sensors of the innate immune system that regulate caspase-1 activation in response to host-derived proteins. Accordingly, NLRP activates apoptosis cascades which contributes to cancer chemoresistance. Overall, TLR/NF-κB/NLRP play critical roles in the development of cancer chemoresistance mediated by the attenuation of apoptosis, inflammation, oxidative stress, and autophagy. As shown in **Figure 2**, the immune system is also cross-talked with dysregulated major signaling pathways of chemoresistance.

## Methodology for Literature Search on the Effect of Phytochemicals on Chemotherapy and Immunotherapy Resistance

We have performed a systematic review on vital mechanisms and the therapeutic potential of plant secondary metabolites in combating cancer chemoresistance utilizing the PRISMA guideline. Scholarly electronic databases, including Scopus, Science Direct, Cochrane, and PubMed, were used for the literature search. The search included all English language articles through October 30, 2021. The following keywords were used for the search: chemoresistance [full text] OR (cancer OR malignancy OR neoplasm OR melanoma OR leukemia OR carcinoma) [title/abstract] AND (nuclear factor kappa* OR NF-κB OR toll-like* OR nod-like receptor* OR NLRP) AND (chemotherapy OR chemoresistance OR immunotherapy OR chemo-therapy OR chemo-resistance OR immune-therapy) [title/abstract] AND (herb OR plant OR natural product OR secondary metabolite OR polyphenol* OR terpen* OR alkaloid* OR flavonoid* OR glucosinolate* OR coumarin*). Two independent authors (S.F. and S.Z.M.) designed and applied the search strategy, which was finalized by the senior author (A.B.).

**Figure 3:**
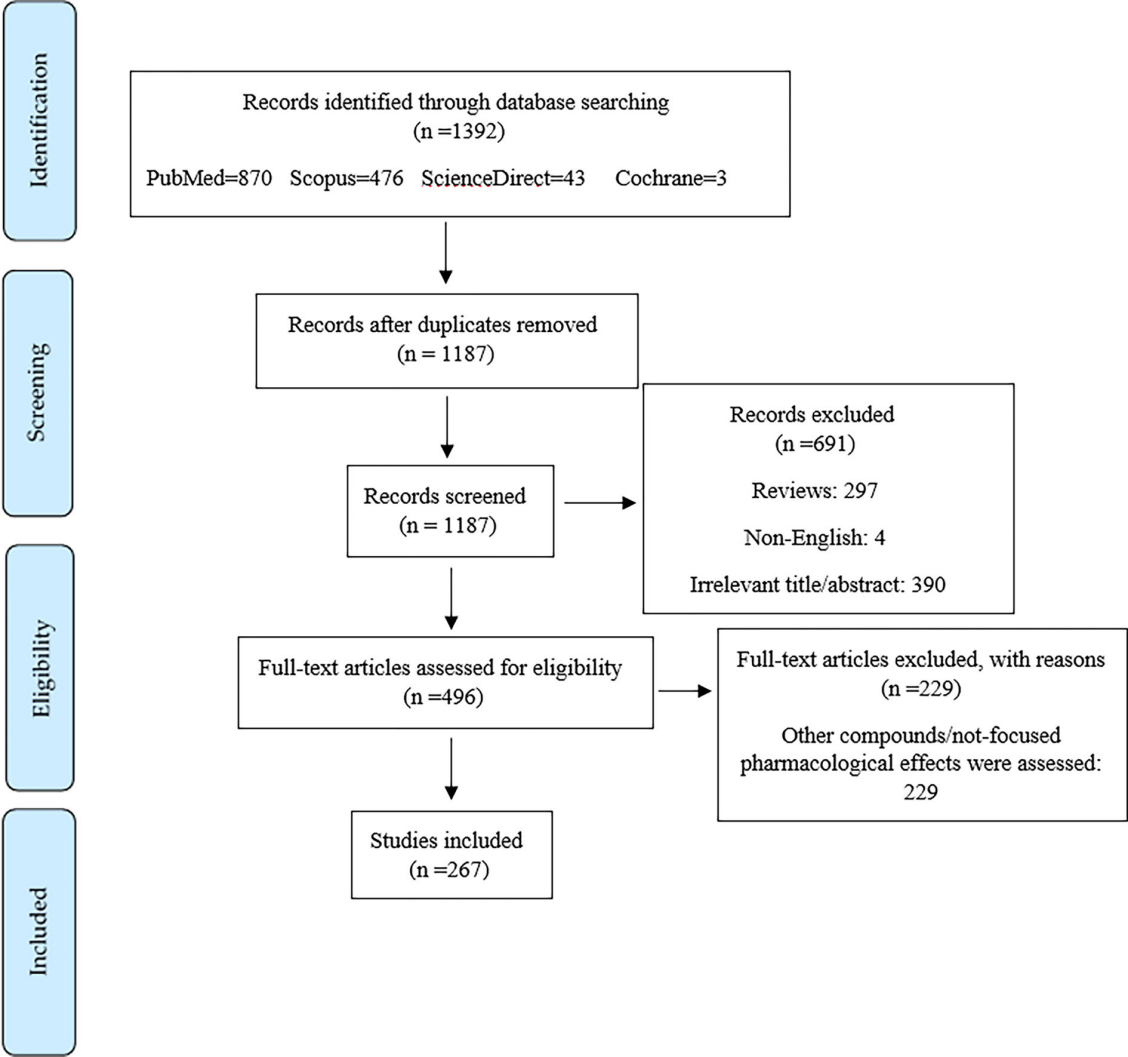
PRISMA flowchart on the process of literature search and selection of relevant studies.

Of the initial 1392 articles, 205 articles were excluded due to duplicated results, 297 articles were excluded as they were review articles, 691 articles were excluded according to their title/abstract, 229 articles were excluded according to their full text information, and 4 articles were omitted since they were not in English. Ultimately, 267 articles were included in this systematic review. **Figure 3** depicts the PRISMA flowchart, which displays the literature search process and selection of relevant studies.

## Multi-Targeting Phytochemicals in Cancer Therapy

Plant secondary metabolites are potential modulators of multiple dysregulated pathways due to their various pharmacological properties, including antioxidant, anti-inflammatory, and anticancer effects (78, 79). An increasing number of pre-clinical and clinical studies have shown that chemopreventive agents may regulate the aforementioned dysregulated signaling pathways, such as TLR, NF-κB, and NLRP, thereby preventing or treating multiple cancer complications (16). Considering the critical role of TLR/NF-κB/NLRP in the progression of chemotherapy and immunotherapy resistance, discovering multi-targeting therapeutic agents could assist in combating cancer chemoresistance and immunoresistance. Several reports have addressed the potential of phytochemicals in the attenuation of TLR/NF-κB/NLRP. Therefore, phenolic compounds, alkaloids, terpenes/terpenoids, and sulfur compounds have been proposed as potential agents in the prevention and treatment of chemoresistance and immunoresistance.

## Phytochemicals Augment Chemotherapy and Immunotherapy Through TLR/NF-κB/NLRP Pathway

Phytochemicals may be used as alternative anticancer agents to prevent chemoresistance. This is made possible by surpassing the resistance barrier in multiple pathways, leading to increased effectiveness. Another benefit of utilizing phytochemicals is that they lower the dose frequency and thus the toxicity of chemotherapeutic agents. The principal mechanism of these phytochemical effects is through the inhibition or overexpression of certain proteins, enzymes, and other cancer cell metabolites.

### Phenolic Compounds

**Figure 4:**
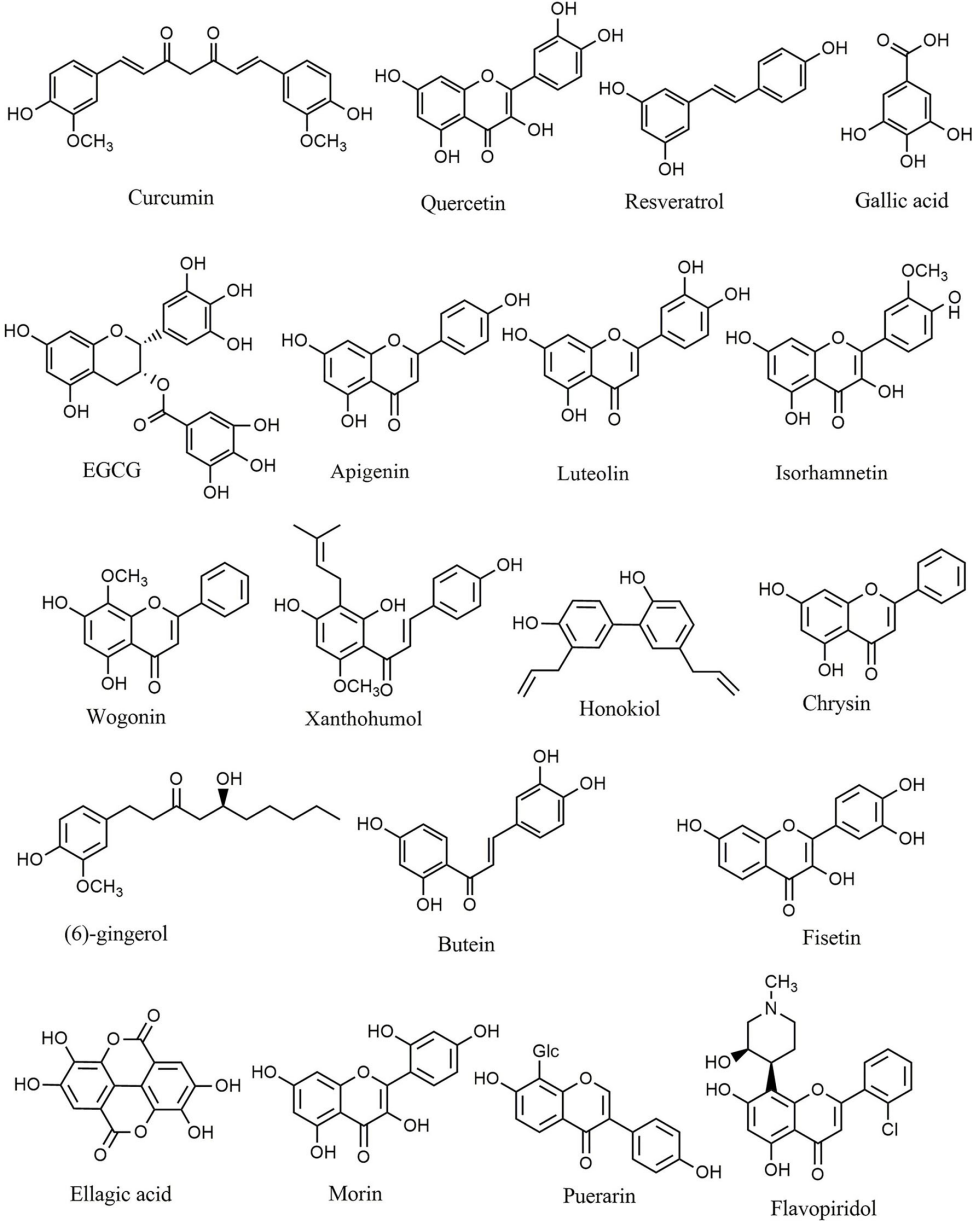
Chemical structures of selected phenolic compounds that modulate the TLR/NF-κB/NLRP signaling in cancer.

Natural polyphenols are an important class of plant secondary metabolites that play an active role against different types of stress. The considerable volume of reported data proposed that diets rich in phenolic compounds could decrease the incidence of several cancers. Curcumin (**Figure 4**) is a well-known phytochemical with several important biological activities, including anticarcinogenic, neuroprotective, anti-inflammatory, and anti-SARS-CoV-2 effects (6, 13, 80–85). Curcumin suppressed the proliferation of MHCC97H liver cancer cells *in vitro* by promoting the formation of intracellular ROS, increasing apoptosis, and activating caspase-3, caspase-8, and TLR4/MyD-88 signaling (86). Furthermore, suppression of HSP70/TLR4 signaling was reported as another anticancer mechanism of curcumin in liver cancer (87). Curcumin also inhibited the growth of liver cancer *in vivo* and *in vitro via* diminished expression of inflammatory factors, such as cyclooxygenase-2 (COX-2), prostaglandin E2, IL-1β, and IL-6, as well as inhibition of the TLR4/NF-κB signaling pathway. Moreover, curcumin reduced VEGF, granulocyte-colony-stimulating factor (G-CSF), and granulocyte−macrophage colony-stimulating factor (GM-CSF) (88). Additionally, curcumin decreased the migration and proliferation of non-small-cell lung cancer cell (NSCLC) cells *via* interfering with EGFR and TLR4/MyD88 pathways and increasing cell cycle arrest in the G2/M phase (89). Treatment with curcumin has also decreased the viability of MCF-7 and MDA-MB-231 breast cancer cells, activated TLR4/TRIF/IRF-3 signaling through the inhibition of IFN-α/β, and reduced the expression of TLR4 and IRF-3 (90). In a similar study, curcumin reduced cell proliferation, inhibited NF-κB, downregulated cyclin D1, and modulated expression of TLR3 in head and neck squamous cell carcinomas (HNSCC) (91). Other reported antitumor mechanisms of curcumin include inhibition of NF-κB, cell cycle arrest, upregulation of E-cadherin, and modulation of Wnt/β-catenin signaling (92, 93). Deng et al. investigated the synergistic inflammatory and immunomodulatory activity of curcumin in combination with total ginsenosides for the treatment of HepG2 liver cancer cells in a BALB/c mice model. The results demonstrated that combination treatment inhibited the growth of liver cancer, reduced the expression of PD-L1, and suppressed the TLR4/NF-κB and NF-κB/matrix metalloproteinase-9 (MMP-9) signaling pathways (94). Curcumin exerts antineoplastic effects on various *in vitro* and *in vivo* cancer models, including lung (95), colorectal (96–98), bladder (99, 100), pancreatic (101, 102), and breast (103) cancers *via* interfering with the expression of TNF-α, HIF-1α, COX-2, VEGF, NF-κB, Axin2, IL-10, IL-8, and IL-6 and participating in the PI3K/Akt/mTOR/NF-κB/Wnt pathway. Additionally, curcumin promoted apoptosis, inhibited NF-κB, MMP-9, MMP-2, and MAPK, and activated sirtuin 1 (SIRT1) in HNSCC, osteoclastoma, and monocytic leukemia SHI-1 cancer cells (104–107). The known plant flavonoid quercetin is widely distributed in many vegetables, seeds, leaves, and grains and shows promising biological properties. Quercetin (**Figure 4**) and curcumin act synergistically together to promote apoptosis in K562 leukemia cells by interfering with the p53, TGF-α, and NF-κB pathways (108).

Resveratrol belongs to the stilbenoid group of polyphenols that exhibit high antioxidant and antitumor potential, which can be found in more than 70 plant species, particularly in grapes’ seeds and skin. Resveratrol (**Figure 4**) and quercetin potentiated the antineoplastic activity of curcumin in myeloid, adenocarcinoma, and HNSCC cells (109–111). Resveratrol reduced dimethylbenz(a)anthracene (DMBA) induced cutaneous carcinogenesis both *in vitro* and *in vivo via* inhibition of angiogenesis. It was reported that TLR4 is a significant mediator involved in the chemoprevention achieved by resveratrol (112). Moreover, resveratrol diminished the inflammatory responses induced by lipopolysaccharide in SW480 and Caco-2 colon cancer cell lines by reducing the activation, expression, and production of inducible NO synthase (iNOS), mRNA, TLR4, and NF-κB (113). Furthermore, resveratrol suppressed the activity of COX, AMP-activated protein kinase (AMPK), PI3K/Akt/NF-κB pathway, DNA methyltransferase, and CYP1A1 in acute myeloid leukemia (AML), colon, and pancreatic cancer cells (114–116). In similar studies, resveratrol exerted substantial antineoplastic activity against multiple cancer cell lines, including melanoma (117), lung (118, 119), glioblastoma (120), head, neck (109), hepatocellular (121), colorectal (122), and breast (123) cancer cells by interfering with dicer-like 1 (DCL1)/translationally controlled tumor protein (TCTP), Akt/NF-κB, retinoblastoma protein (pRB), VEGF, AMPK, and p21^Waf1/Cip1^ signaling pathways. Luteoloside (known as Cynaroside), 7-O-glucoside of luteolin, is a flavone agent that inhibited metastasis and proliferation of SNU-449, Hep3B, and mouse lung cancer cells through inhibition of caspase-1, NLRP3, and IL-1β (124).

Gallic acid is a natural antioxidant found in various fruits and tea leaves that belongs to the polyphenolic class of secondary metabolites. Various pharmacological effects of gallic acid include antioxidant, anti-inflammatory, and antineoplastic activities. There have been multiple studies which report that gallic acid (**Figure 4**) inhibited the progression of T24 and AGS gastric cancer cells *via* suppression of PI3K/Akt/NF-κB signaling and promotion of mitochondrial dysfunction (125, 126). Furthermore, quercetin inhibited the invasion and migration of Caco-2 cells *via* regulation of the TLR4/NF-κB pathway and decreasing MMP-2 and MMP-9 (127). In a similar study, inhibition of NF-κB, p53 induction, apoptosis, and cell cycle arrest were reported as the primary anticancer mechanisms of quercetin against the HeLa cervical cancer cell line (128). Additionally, quercetin showed antitumor activity against lung (A549 and H460) (129, 130), prostate (PC3 and LNCaP) (131), breast (MCF-7) (132), and oral SCC (133) cancer cells *via* induction of apoptosis and downregulation of IL-6/STAT-3 and NF-κB. The results demonstrated that combination of quercetin with chrysin suppressed the migration and invasion of nickel via downregulation of TLR4/NF-κB signaling in human lung cancer cells *in vitro* (134). Octyl gallate exerted significant efficacy against heat shock protein 90α (HSP90α) levels, eHSP90α–TLR4 ligation, M2-macrophages, and tumor growth in a pancreatic ductal adenocarcinoma mouse model (135).

Moreover, several studies have been performed to investigate the various biological effects of epigallocatechin 3-gallate (EGCG), a powerful polyphenolic isolated from green tea. EGCG showed significant anti-inflammatory, antioxidant, anticancer, and neuroprotective potential in different studies. Treatment with EGCG (**Figure 4**) downregulated the expression of NLRP1, caspase-1, and IL-1β in the melanoma cell lines HS294T and 1205Lu (136). The main antineoplastic mechanisms of EGCG includes inhibition of TNF-α and tissue factor expression (137), activation of forkhead box O3 (138), downregulation of Her-2/Neu signaling (139), decreased expression of IL-1RI (140), and modulation of MMP-2 activity (141). Furthermore, EGCG induced apoptosis and suppressed cancer cell proliferation in nasopharyngeal (142), bladder (143), hepatocellular (144), breast (145), and colon (146) cancers.

Similarly, apigenin is another polyphenolic substance with significant anticancer potential *via* modulating different signaling pathways *in vitro* and *in vivo*. Treatment with apigenin induced apoptosis, suppressed glycogen synthase kinase-3 (GSK-3)/NF-κB, and downregulated Bcl-xL, CCL2, CXCL-8, IL1A, Bcl-2, and VEGF in pancreatic (PANC-1 and BxPC-3) (147), prostate (PC-3) (148, 149), and breast (MDA-MB-231) cancer cells (150), as well as the athymic nu/nu nude (151) and TRAMP mice (152) cancer models. In addition to apigenin, luteolin (**Figure 4**) has significant anticancer properties. Luteolin interfered with the PI3K/Akt/NF-κB/Snail and MAPK pathways in the gastric adenocarcinoma cell line CRL-1739, the lung cancer cell line A549, and the AML cell line THP-1 (153–155). Additionally, luteolin 8-C-b-fucopyranoside suppressed secretion of MMP-9, IL-8, ERK/NF-κB, and ERK/AP-1 signaling in MCF-7 cancer cells *in vitro* (156). The *in silico* evaluations also showed that luteolin and other plant-derived secondary metabolites (e.g., myricetin, quercetin, apigenin, and baicalein) displayed anticancer properties *via* the estrogen receptor-α (157).

Isorhamnetin (**Figure 4**) is another polyphenolic compound that induced apoptosis and inhibited proliferation of lung (158) and breast (159) cancer cells by interfering with IL-13, NF-κB, MAPK, and Akt signaling. Similarly, wogonin (**Figure 4**) promoted apoptosis and suppressed the invasion and proliferation of chronic lymphocytic leukemia and the liver cancer cell lines Bel7402 and HepG2 by interfering with ERK/AKT, NF-κB/Bcl−2, and EGFR signaling pathways (160, 161). Another polyphenolic structure, xanthohumol (**Figure 4**), appears to have anticancer properties *via* significantly suppressing the angiogenesis, proliferation, and production of inflammatory mediators in breast cancer xenografts (162). Xanthohumol also reduced the expression of CXCR4 and inhibited cancer cell invasion (163). Similarly, p-hydroxycinnamic acid facilitated cell cycle arrest and suppressed the growth and migration of MDA-MB-231 cells *via* downregulation of NF-κB (164). The flavonoid wogonoside demonstrated anticancer effects *in vitro* against MCF7 and MDA-MB-231 cancer cells through inhibition of migration, invasion, and TRAF-2/TRAF-4 expression (165). In similar studies, inhibiting COX-2, EGFR, NF-κB, and the ERK pathway is the main anticancer mechanism of scutellarein in A549 cells (166). Likewise, hydroxysafflor yellow A (167), rosmarinic acid (168, 169), and magnolol (170) diminished the progression of hepatocellular carcinoma *in vitro* and *in vivo* by suppressing ERK/MAPK, ERK/NF-κB, and NF-κB signaling. Lung cancer cells treated with hexamethoxy flavanone-o-[rhamnopyranosyl-(1→4)-rhamnopyranoside, a flavonoid glycoside compound isolated from *Murraya paniculata* (171), hesperetin (172), honokiol (**Figure 4**) (173, 174), and inotilone (174) had higher levels of apoptosis-related mediators and attenuated activity of EGFR, PI3K/Akt/MAPK, and STAT3/NF-κB/COX-2 signaling pathways. Investigation into the effects of eupatilin (175, 176), polysaccharide krestin (177), tilianin (178), silibinin (179–181), chrysin (**Figure 4**) (182–186), (6)-gingerol (**Figure 4**) (187), and butein (**Figure 4**) (188) against gastric, breast, ovarian, pharyngeal squamous, prostate, renal, myeloid leukemia, and T cell leukemia/lymphoma cancer cells *in vitro* and *in vivo* demonstrated that these polyphenolics exert significant effects *via* attenuation of angiogenesis, Akt, tumor growth, AP-1, NF-κB, and TLR4.

Fisetin (**Figure 4**) (189–194), gallotannin (195), astragalin (196–198), ellagic acid (**Figure 4**) (199–201), morin (**Figure 4**) (202, 203), flavopiridol (**Figure 4**) (204), puerarin (**Figure 4**) (205), icariin (206), acteoside (207), and acacetin (208, 209) are some of the other polyphenolic agents that inhibited the proliferation and invasion of breast, prostate, hepatocellular, myeloma, and colon cancer cells *via* increased apoptosis and inhibition of TNF-α, iNOS, NF-κB, COX-2, JAK/STAT3, Akt, and IL-6 mediators and signaling pathways.

In further studies, eriodictyol (210, 211), calycosin (212), cudraflavone B (213), protocatechualdehyde (214), and naringin (215) exerted significant antitumor effects against several cancers including breast (MDA-MB-231), glioblastoma (A172, CHG-5, and U87 MG), ovarian (SKOV3), and liver (HepG2) cancer cells by promoting senescence, apoptosis, and interfering with GSK-3β, TGF-β1, SMAD2/3, SLUG, vimentin, β-catenin, NF-κB, COX-2, and cyclin D1, amongst other enzymes and signaling pathways.

In summary, polyphenols play a significant role in the prevention and treatment of cancer. Curcumin, apigenin, quercetin, and resveratrol are the most important polyphenols with reported information on their mechanisms of action and clinical trials. In several reported studies, polyphenols can interfere with a variety of anticancer pathways, including TLR/NF-κB/NLRP and interconnected pathways. Consequently, polyphenols could be considered promising treatment options in conjunction with other cancer treatment strategies. **Table 1** provides the various anticancer phenolic compounds that interfere with the TLR/NF-κB/NLRP pathway to combat chemoresistance.

**Table 1:** Anticancer phenolic compounds interfering with the TLR/NF-κB/NLRP pathway and cross-talked mediators against chemoresistance.

Compound	Types of study	Cell line(s)/tumor model(s)	Mechanisms of action	References
Curcumin	*In vitro*	Hepatocellular carcinoma cells (MHCC97H)	↑ROS/TLR4/caspase pathway; ↓cell proliferation; ↑apoptosis; ↑ROS formation; ↑caspase-8; ↑caspase-3	(86)
	*In vitro*	Hepatocellular carcinoma cells (HepG2)	↓TLR4; ↓proliferation; ↓invasion; ⟂ S phase cell cycle; ↑apoptosis; ↓HSP70; ↓EHSP70	(87)
	*In vitro* and *in vivo*	Human liver cancer cells (HepG2, MHCC−97H, and huh−7); Xenograft model mice	↓TLR4/NF−κB; ↓VEGF; ↓COX-2; ↓PGE2; ↓IL−1β; ↓IL−6	(88)
	*In vitro*	Lung adenocarcinoma cells (H226, NSCLC, NCI−A549, NCI−H226)	↓TLR4/MyD88; ↓migration; ↓proliferation; ↓EGFR; ⟂G2/M phase cell cycle	(89)
	*In vitro*	Breast cancer cells (MDA-MB-231, MCF-7)	↑TLR4/TRIF/IRF-3; ↓IFN-α/β; ↓TLR4; ↓IRF-3	(90)
	*In vitro*	Head and neck squamous cell carcinomas (HNSCC)	↓NF-κB; ↓TLR3; ↓proliferation; ↓cyclin D1	(91)
	*In vitro*	Nasopharyngeal carcinoma cells (HK1 and HONE1)	↓NF-κB; ↑E-Cadherin	(93)
	*In vitro*	Human mantle cell lymphoma	↓NF-κB; ⟂G1/S phases cell cycle; ↓IKK; ↓phosphorylation of IκBα and p65	(92)
	*In vitro* and *in vivo*	Human liver cancer cell (HepG2); Male nude mice models of liver cancer	↓TLR4/NF-κB; ↓Tregs; ↓MMP-9; ↓PD-L1; ↓NF-κB/MMP-9	(94)
	*In vitro* and *in vivo*	Lung cancer cell (A549); BALB/c nude mice	↓NF-κB; ↓tumor growth; ↓Notch-1; ↓HIF-1; ↓VEGF	(95)
	*In vivo*	C57BL/6 male mice	↓Wnt/β−catenin; ↓Axin2; ↓tumor number; ↓Tumor size; ↓β−catenin; ↓cell proliferation	(97)
	*In vivo*	A/J mice model	↓NF-κB; ↓Cell proliferation; ↓PI3K/Akt/mTOR; ↓AMPK	(96)
	*In vitro*	Colorectal carcinoma cell (HCT−116)	↓NF-κB	(98)
	*In vitro*	Albino rats’ model of bladder cancer	↓NF-κB; ↓Bcl-2; ↓IL-6; ↓P65	(99)
	*In vitro* and *in vivo*	Human bladder cancer cell (T24, UMUC3); Male nude mice	↓NF-κB p65; ↓IKKκ/NF-κB/COX-2; ↓COX-2 expression; ⟂ G2/M phase cell cycle; ↓CDK1; ↓cyclin A; ↓cyclin B; ↓cyclin D1;	(100)
	Clinical trials	Patients with advanced pancreatic cancer	↓NF-κB; ↓COX-2; ↓pSTAT 3	(102)
	*In vitro*	Pancreatic cancer cells (Su86.86, PL8, Panc1, BxPC3, MiaPaca2, E3LZ10.7, and Capan1, PL5)	↓NF-κB; ↑apoptosis; ↓IL-6; ↓IL-8; ↓TNF-α	(101)
	*In vitro*	Breast cancer cell (MCF-7)	↓NF-κB; ↓MMP; ↓AP-1; ↓PKCα; ↓MAPK	(103)
	*In vitro*	HNSCC cell (FaDu and Cal27)	↓NF-κB; ↑caspase-9; ↑caspase-8; ↑ATM/CHK2	(105)
	*In vitro*	Monocytic leukemia cell (SHI-1)	↓NF-κB; ↓Bcl-2; ↓ERK; ↑p38 MAPK; ↑JNK; ↑caspase-3; ↓MMP-2; ↓MMP-9	(106)
	*In vitro and in vivo*	SCID mice; Acute monocytic leukemia SHI-1 cells	↓NF-κB and ERK; ↓PCNA; ↑cleaved caspase-3; ↑p38 and JNK; ↓MMP-2 and MMP-9	(107)
	*In vitro*	Human osteoclastoma cell (GCT cells)	↑Apoptosis; ↓NF-κB; ↑caspase-3; ↓MMP-9; ↑JNK	(104)
Curcumin & Quercetin	*In vitro*	Chronic myeloid leukemia (CML) (K562)	↓NF-κB; ↑apoptosis; ↓IFN-c; ↓AKT1; ↓CDKN1B; ↑p21 ^Waf1/cip1^; ↑FasL; ↑Fas	(108)
Curcumin & Quercetin	*In vitro*	CML cell (K562/CCL-243)	↑BTG2; ↑CDKN1A; ↑FAS; ↓CDKN1B; ↓AKT1; ↓IFN-c; ↑p21 ^Waf1/cip1^	(111)
Curcumin & Quercetin	*In vitro*	Human melanoma cell (A375)	↓Cell proliferation; ↓Wnt/β-catenin; ↓DVL2; ↓cyclin D1; ↓COX2; ↓Axin2; ↓BCL2; ↑caspase 3/7; ↑PARP cleavage	(110)
Curcumin & Resveratrol	*In vitro* and *in vivo*	HNSCC; BALB/c mice	↓NF-κB; ↑PARP-1 cleavage; ↑Bax/Bcl-2 ratio; ↓ERK1; ↓ERK2 phosphorylation	(109)
Resveratrol	*In vivo*	Female C3H/HeN mice	↓Angiogenesis; ↓MMP-2; ↓MMP-9; ↑IL-12	(112)
	*In vitro*	Human colon adenocarcinoma cells (SW480, Caco-2)	↓NF-κB; ↓TLR4 expression; ↓NO; ↓iNOS;	(113)
	*In vitro*	Colon cancer cell (HCT-116 and SW-480)	↓NF-κB; ↓CYP1A1 activity; ↓DNA methyltransferase; ↓COX; ↓cytokine production; ↓AMPK	(116)
	*In vitro*	Pancreatic cancer cell (BxPC-3 and Panc-1)	↓PI3K/Akt/NF-κB; ↓cell proliferation; ↓cell migration; ↓cell invasion; ↓p-Akt; ↓p-NF-κB	(115)
	*In vitro*	Acute myeloid leukemia cell (OCI/AML3, OCIM2)	↓NF-κB; ↓cell proliferation; ⟂S phase cell cycle; ↑apoptosis	(114)
	*In vitro*	Melanoma cell line (LU1205, LU1205, 1205lu, FEMX, WM35, WM793, HHMSX, OM431, WM9, LOX)	↓NF-κB; ↓STAT3; ↓cFLIP; ↓Bcl-XL	(117)
	*In vitro*	Human lung carcinoma (A549, HCC-15)	↓NF-κB; ↓p-Akt; ↓Bcl-2; ↓Bcl-XL	(119)
	*In vitro*	Human lung carcinoma (A549)	↓NF-κB; ↓pRB; ↓p21^Waf1/Cip1^; ↑Apoptosis;	(118)
	*In vitro*	Glioblastoma cell (T98G)	↓NF-κB; ↓MGMT	(120)
	*In vitro* and *in vivo*	BALB/c mice; HNSCC (CAL-27, SCC-15)	↓NF-κB; ↑PARP-1 cleavage; ↑Bax/Bcl-2 ratio; ↓ERK1; ↓ERK2 phosphorylation	(109)
	*In vitro* and *in vivo*	Hepatocellular carcinoma cells HepG2 cells; Xenograft models	↓NF-κB; ↓VEGF	(121)
	*In vitro*	Colorectal cancer cell (HCT116/L-OHP)	↓NF-κB; ↓cAMP; ↓MDR1; ↓NF-κB; ↓p-IκBα; ↓MDR1; ↑pAMPK	(122)
	*In vitro*	Human liver cancer cell (HepG2); Breast cancer cell (MDA-MB-231)	↓JNK/NF-κB; ↑DLC1 and ↓TCTP; ↓N-WASP; ↑Cdc42	(123)
Luteoloside	*In vitro and in vivo*	Mouse lung metastasis model; Hepatocellular carcinoma cell (SNU-449, Hep3B)	↓NLRP3; ↓cleavage of caspase-1; ↓IL-1β	(124)
Gallic Acid	*In vitro*	Human bladder cancer cells (T24)	↓PI3K/Akt/NF-κB; ↑apoptosis; ↑cleaved caspase-3; ↑Bax, P53; ↑cyt c; ↓Bcl-2; ↓p-PI3K; ↓pAkt; ↓p-IkBa; ↓p-IKKa; ↓p-NF-κB p65	(126)
	*In vitro*	Human gastric cancer cell (AGS)	↓NF-κB; ↓MMP-2/9	(125)
Quercetin	*In vitro*	Human colon adenocarcinoma cell (Caco-2)	↓TLR4/NF-κB; ↓migration; ↓Invasion; ↓MMP−2; ↓MMP−9	(127)
	*In vitro*	Human cervical cancer cell (HeLa)	↓NF-κB; ↑p53; ↑apoptosis; ⟂ G2/M cell cycle arrest; ↑Bcl-2; ↑cyt c; ↑Apaf-1	(128)
	*In vitro*	Lung cancer cell (A549)	↓NF-κB; ↓IL-6/STAT-3; ↓IL-6; ↑Apoptosis	(130)
	*In vitro*	Lung cancer cells (H460)	↓NF-κB; ↑apoptosis; ↑TRAILR; ↑caspase-10; ↑DFF45; ↑TNFR 1; ↑FAS; ↑DNA damage	(129)
	*In vitro*	Breast cancer cells (MDA-MB-231, MCF-7)	↑Apoptosis; ↓Hsp27, Hsp70 and Hsp90;	(132)
	*In vitro*	Oral squamous cell carcinoma (OSCC)	↓NF-κB; ↓tumor incidence; ↑apoptosis; ↓Bcl-2; ↓Bax	(133)
	*In vitro*	Prostate cancer cells (PC3, LNCaP)	↓NF-κB; ↓PI3K/Akt; ↓MAPK/ERK; ↑apoptosis; ⟂G1 phase cell cycle arrest; ↓P38; ↓ABCG2	(131)
Quercetin and chrysin	*In vitro*	Human lung adenocarcinoma cell (A549)	↓TLR4/NF-κB	(134)
Octyl Gallate	*In vitro* and *in vivo*	Adenocarcinoma cell (AsPC-1, Panc 02); Monocytic leukemia cell (THP-1); Male C57BL/6 mice	↓EHSP90α–TLR4 ligation; ↓HSP90α level; ↓M2-macrophages; ↓tumor growth	(135)
EGCG	*In vitro*	Human melanoma cell (1205Lu and HS294T)	↓NLRP1; ↓caspase-1; ↓IL-1β	(136)
	*In vitro*	Monocytic leukemia cell (THP-1)	↓TLR4; ↓NF-κB; ↓tissue factor; ↓TNF-α; ↓p-p38; ↓ERK1/2; ↓JNK	(137)
	*In vitro*	Breast cancer cell (NF639)	↓NF-κB; ↓colony growth; ↓Cell invasion; ↓protein kinase CK2	(138)
	*In vitro*	Breast cancer cell (SMF, NF639)	↓NF-κB; ↓Her-2/Neu signaling; ↓Cell growth; ↓PI3K; ↓Akt; ↓cell proliferation	(139)
	*In vitro*	Pancreatic adenocarcinoma cell (Colo357)	↓NF-κB; ↓IL-1RI; ↑apoptosis	(140)
	*In vitro* and *in vivo*	Breast cancer cell (MCF-7)	↓NF-κB; ↓MMP-2; ↓FAK; ↓MT1-MMP; ↓VEGF	(141)
	*In vitro* and *in vivo*	Bladder cancer cells (SW780); Xenograft mice	↓NF-κB; ↓MMP-9	(143)
	*In vitro* and *in vivo*	Nude mice; Hepatocellular carcinoma cells (HepG2, Huh-7, PLC/PRF/5)	↓NF-κB; ↓Bcl-2; ↓Bcl-XL	(144)
	*In vitro* and *in vivo*	Breast cancer cell (4T1); BALB/c mice	↓MDSCs; ↓Arg-1/iNOS/Nox2/NF-κB/STAT3	(145)
	*In vitro*	Human colorectal cancer cells (RKO, HT-29, HCT-116)	↑NF-κB-p65; ⟂ G2/M and G1 phases cell cycle; ↑p21; ↑p53; ↓Survivin	(146)
Apigenin	*In vitro*	Human pancreatic cancer cell (PANC-1, BxPC-3)	↑Apoptosis; ↓GSK-3/NF-κB; ⟂G2/M phase cell cycle	(147)
	*In vitro*	Cancer stem cells; Prostate cancer cell (PC3)	↓PI3K/Akt/NF-κB; ↑p21; ↑p27; ↑caspases-8, caspase-3; ↑TNF-α; ↑Bax; ↑cyt c; ↓MMP-2, -9; p105/p50; ↓PI3K	(149)
	*In vitro*	Prostate carcinoma cell (PC-3)	↓NF-κB; ↑apoptosis	(148)
	*In vitro*	Breast cancer cell (MDA-MB-231)	↓CCL2; ↓CXCL-8; ↓IL1A	(150)
	*In vivo*	Xenograft athymic nu/nu nude mice	↓Cell proliferation; ↓Her2/neu; ↓VEGF	(151)
	*In vivo*	TRAMP mice	↓NF-κB; ↓tumor volumes; ↓Phosphorylation IκBα; ↓IKK; ↓Bcl2; ↓Bcl-XL; ↓cyclin D1; ↓COX-2; ↓VEGF	(152)
Luteolin	*In vitro*	Lung adenocarcinoma cell (A549)	↓NF-κB–Snail; ↓E-cadherin; ↓PI3K/Akt/IκBa–	(153)
	*In vitro*	Monocytic leukemia cell (THP-1)	↓NF-κB; ↓MAPK; ↑HO-1; ↑pAkt; ↓IL-6; ↓IL-8; ↓SICAM-1; ↓MCP-1	(154)
	*In vitro*	Gastric cancer cell (CRL−1739)	↑NF−κB; ↑IL−8; ↑IL−10	(155)
Luteolin 8-C-b-fucopyranoside	*In vitro*	Breast cancer cell (MCF7)	↓ERK/NF-κB; ↓MMP-9; ↓IL-8; ↓ERK/AP-1	(156)
Isorhamnetin	*In vitro*	Lung adenocarcinoma cell (A549)	↓NF-κBp65; ↑apoptosis	(158)
	*In vitro*	Breast cancer cell (MCF7 or MDA-MB-468)	↓Cell proliferation; ↑Apoptosis; ↓Akt/mTOR; ↓MEK/ERK phosphorylation	(159)
Wogonin	*In vitro* and *in vivo*	C57BL/6 mice; Lymphocytic leukemia cell (TCL1)	↓NF-κB	(161)
	*In vitro*	Human hepatocellular cancer cells (Bel7402, HepG2)	↓NF−κB/Bcl−2; ↓cell proliferation; ↓cell invasion; ↑Apoptosis; ↓EGFR; ↓ERK/AKT; ↓cyclin D1	(160)
Xanthohumol	*In vitro* and *in vivo*	Breast cancer cell (MCF-7); Xenografts nude mice	↓NF-κB; ↓angiogenesis; ↓Cell proliferation	(162)
	*In vitro*	Breast cancer cells (MDA-MB-231, MCF-7); Colorectal cancer cells (HCT8, HCT116); Pancreatic carcinoma cell (Panc-1)	↓CXCR4; ↓cell invasion	(163)
P-hydroxycinnamic acid	*In vitro*	Breast cancer cells (MDA-MB-231)	↓NF-κB; ⟂G1 phase cell cycle; ⟂G2/M phase cell cycle	(164)
Wogonoside	*In vitro*	Breast cancer cells (MDA-MB-231)	↓NF-κB; ↓cell invasion; ↓cell migration; ↓TNF-α; ↓TRAF-2; ↓TRAF-4; ↓Twist1; ↓MMP-9; ↓MMP-2; ↓vimentin	(165)
Scutellarein	*In vitro*	Lung adenocarcinoma cell (A549)	↓NF-κB; ↓COX-2; ↓EGFR; ↓ERK	(166)
Hydroxysafflor yellow A	*In vitro* and *in vivo*	Murine hepatoma cell (H22); Male Kunming mice	↓NF-κB; ↓Angiogenesis; ↓ERK/MAPK; ↓cyclinD1; ↓c-myc; ↓c-Fos	(167)
Rosmarinic acid	*In vitro* and *in vivo*	Male Kunming mice; Hepatocellular carcinoma cell (H22)	↓Tumor growth; ↓IL-6; ↓IL-10; ↓STAT3; ↑Bax; ↑caspase-3; ↓Bcl-2	(169)
	*In vitro*	Human leukemia cell (U937)	↓NF-κB	(168)
Magnolol	*In vivo*	BALB/cAnN.Cg-Foxn1nu/CrlNarl mice	↓ERK/NF-κB; ↑apoptosis; ↓tumor progression; ↓ MMP-9; ↓ VEGF; ↓ XIAP; ↓cyclin D1	(170)
HMFRR	*In vitro*	Lung adenocarcinoma cell (A549, PC9)	↓STAT3/NF-κB/COX-2; ↓ EGFR/PI3K/Akt; ↓EGFR	(171)
Hesperetin	*In vivo*	Swiss albino mice	↓NF-κB; ↑TNF-α; ↓PCNA; ↓CYP1A1	(172)
Honokiol	*In vitro* and *in vivo*	Orthotopic mouse model; Prostate cancer cell (MiaPaCa, Colo-357)	↓NF-κB; ↓sonic hedgehog; ↓CXCR4	(173)
	*In vitro*	Lung adenocarcinoma cell (A549, H460)	↓C-FLIP; ↓cell migration	(174)
Inotilone	*In vitro* and *in vivo*	Lung adenocarcinoma cell (A549); Mouse Lewis lung carcinoma cell lines; C57BL/6 male mice	↓NF-κB; ↓PI3K/Akt/MAPK; ↓MMP-2/9; ↓IL-8; ↓NO; ↓TNF-α; ↓cell migration; ↓PI3K; ↓PAkt	(174)
Eupatilin	*In vitro*	Gastric cancer cell (MKN-1)	↓NF-κB; ↓tumor invasion; ↑Caspase-3; ↓MMPs	(175)
	*In vitro*	Prostate cancer cell (PC3, LNCaP)	↓PTEN and NF-κB; ↑p53; ↑p21; ↑p27; ⟂G1 phases cell cycle arrest	(176)
Polysaccharide krestin	*In vivo*	Tumor-bearing neu transgenic mice	↓Cell growth; ↑T cell; ↑NK cell	(177)
Tilianin	*In vitro*	Pharyngeal squamous carcinoma cells (FaDu)	↑TLR4/p38/JNK/NF-κB; ↑Apoptosis; ↑TLR4; ↓Bcl-2; ↓Bcl-XL; ↑Bad; ↑Bax; ↑PARP; ↑cyt c; ↑caspase-3	(178)
Silibinin	*In vitro* and *in vivo*	Prostate cancer cells (PC3, WPE-1 NA-22, RWPE-1, WPE-1 NB-14); C57Bl/6 mice	↓NF-κB; ↓MCP-1; ↓CAF; ↓AP-1	(181)
	*In vitro*	Prostate carcinoma cell (DU145)	↓NF-κB	(179)
	*In vitro*	Hepatocellular cancer cells (HepG2); Prostate cancer cells (PC-3)	↓NF-κB; ↓Phospholipase A2	(180)
Chrysin	*In vitro* and *in vivo*	Renal cell carcinoma (RCC); Wistar rats	↓NF-κB; ↓COX-2; ↓iNOS	(182)
	*In vivo*	Swiss albino mice	↓NF-κB; ↓PCNA, ↓COX-2	(185)
	*In vitro*	Lung adenocarcinoma cell (A549); Human cervical cancer cell (HeLa)	↓NF-κB; ↓Mcl-1; ↓pSTAT3; ↓TRAIL; ↑glutathione depletion; ↓STAT3	(183)
	*In vivo*	Wistar rats	↓NF-κB; ↓PCNA; ↓CAT; ↓GPX; ↓SOD	(184)
	*In vitro* and *in vivo*	Cervical cancer cell (HeLa); BALB/c-nu mice	↓NF-κB/Twist Axis	(186)
(6)-Gingerol	*In vitro* and *in vivo*	Myeloid leukemia cells (U937 and K562); Xenograft mice	↓NF-κB; ↑ROS	(187)
Butein	*In vitro* and *in vivo*	T cell leukemia/lymphoma (MT-4, HUT-102); Mice harboring ATLL xenograft	↓NF-κB; ⟂G1 cell cycle arrest; ↓Tumor growth; ↓AP-1; ↓Akt	(188)
Fisetin	*In vitro*	Colorectal cancer cell (HCT116, HT29)	↓Wnt/EGFR/NF-κB; ↑apoptosis; ↓COX2;	(190)
	*In vitro*	Prostate cancer cell (PC-3)	↓PI3K/Akt and JNK; ↓MMP-2/9; ↓MMP-2 and MMP-9	(191)
	*In vitro*	Laryngeal carcinoma cell (TU212)	Regulation of Akt/NF-κB/mTOR and ERK1/2	(194)
	*In vitro*	Pancreatic cancer cell (AsPC-1)	↓NF-κB; ↓DR3; ↓p-NF-κB/p65	(189)
	*In vivo*	Autochthonous Wistar rats	↓NF-κB; apoptosis induction of p53	(192)
Gallotannin	*In vitro* and *in vivo*	Colorectal cancer cell (HCT116, HT29); Xenografts in NOD/SCID mice	↓NF-κB; ↓IL-6; ↓TNFα; ↓IL-1α; ↓VEGFA	(195)
Astragalin	*In vitro* and *in vivo*	Colorectal cancer cell (HCT116); Xenograft in nude mice	↓NF-κB; ↓cell proliferation; ↓cell migration; ⟂G0/G1 phase cell cycle; ↑apoptosis; ↓Bax; ↓Bcl-2; ↓P53; ↓caspase-3,6,7,8,9	(198)
	*In vitro*	Lung cancer cell (A549)	↓NF-κB; ↓ERK-1/2; ↓Akt	(197)
	*In vitro* and *in vivo*	Stomach cancer cell (MKN45); C57/BL female mouse	↑TLR4	(196)
Ellagic acid	*In vivo*	Wistar albino rats	↓NF-κB; ↓TNF-α; ↓IL-6; ↓COX-2; ↓iNOS	(199)
	*In vitro* and *in vivo*	Pancreatic carcinoma (PANC-1); Xenografted mice	↓NF-κB; ⟂G1 phase cell cycle; ↓COX-2; ↑E-cadherin ↓Vimentin	(201)
Morin	*In vivo*	Wistar albino rats	↓NF-κB; ↓TNF-α; ↓IL-6; ↓COX-2; ↓PGE-2	(203)
	*In vitro*	Lung carcinoma cell (A549); Cervical cancer cell (HeLa)	↓NF-κB; ↓cyclin D1; ↓COX-2; ↓MMP-9	(202)
Flavopiridol	*In vitro*	Non-small cell lung carcinoma cell (A549), Colon cancer cells (HCT-116, HCT-15)	↓NF-κB	(204)
Puerarin	*In vitro*	Bladder cancer cell (T24)	↓NF-κB; ↓proliferation; ↑Apoptosis; ↑miR-16; ↓COX-2	(205)
Icariin	*In vitro*	Human myeloma cell (U266)	↓JAK/STAT3; ↓STAT3; ↓VEGF; ↓MMP-9; ↓STAT3	(206)
Acteoside	*In vitro*	Hepatocellular carcinoma cell (HepG2, HuH7)	↓NF-κB	(207)
Acacetin	*In vitro* and *in vivo*	Xenografted mice; Prostate cancer cell (DU145)	↓NF-κB/Akt; ↓XIAP; ↓COX-2	(209)
	*In vitro*	Prostate cancer cell (DU145)	↓NF-κB; ↓p38MAPK; ↓AP-1-binding	(208)
Eriodictyol	*In vitro*	Glioblastoma cells (A172, U87 MG)	↓Cell proliferation; ↓apoptosis; ↓EMT markers; ↓p38 MAPK/GSK-3β/ZEB1	(211)
	*In vitro* and *in vivo*	Glioblastoma cells (CHG-5, U87 MG); Mouse xenograft model	↓PI3K/Akt/NF-κB; ↑apoptosis	(210)
Calycosin	*In vitro*	Hepatocellular carcinoma cell (HepG2)	↓Bcl-2; ↑Bax; ↑Caspase-3; ⟂ G0/G1 phase cell cycle; ↓Akt; ↓TGF-β1; ↓SMAD2/3; ↓SLUG; ↓vimentin	(212)
Cudraflavone B	*In vitro*	OSCC	⟂sub-G1 phase cell cycle; ↑p27	(213)
Protocatechualdehyde	*In vitro*	Breast cancer cell (MCF-7, MDA-MB-231)	↓NF-κB; ↓GSK-3β; ↓β-catenin; ↓cyclin D1	(214)
Naringin	*In vitro*	Ovarian cancer cells (SKOV3/DDP)	↓NF-κB; ↓COX-2	(215)

### Terpenes and Terpenoids

**Figure 5:**
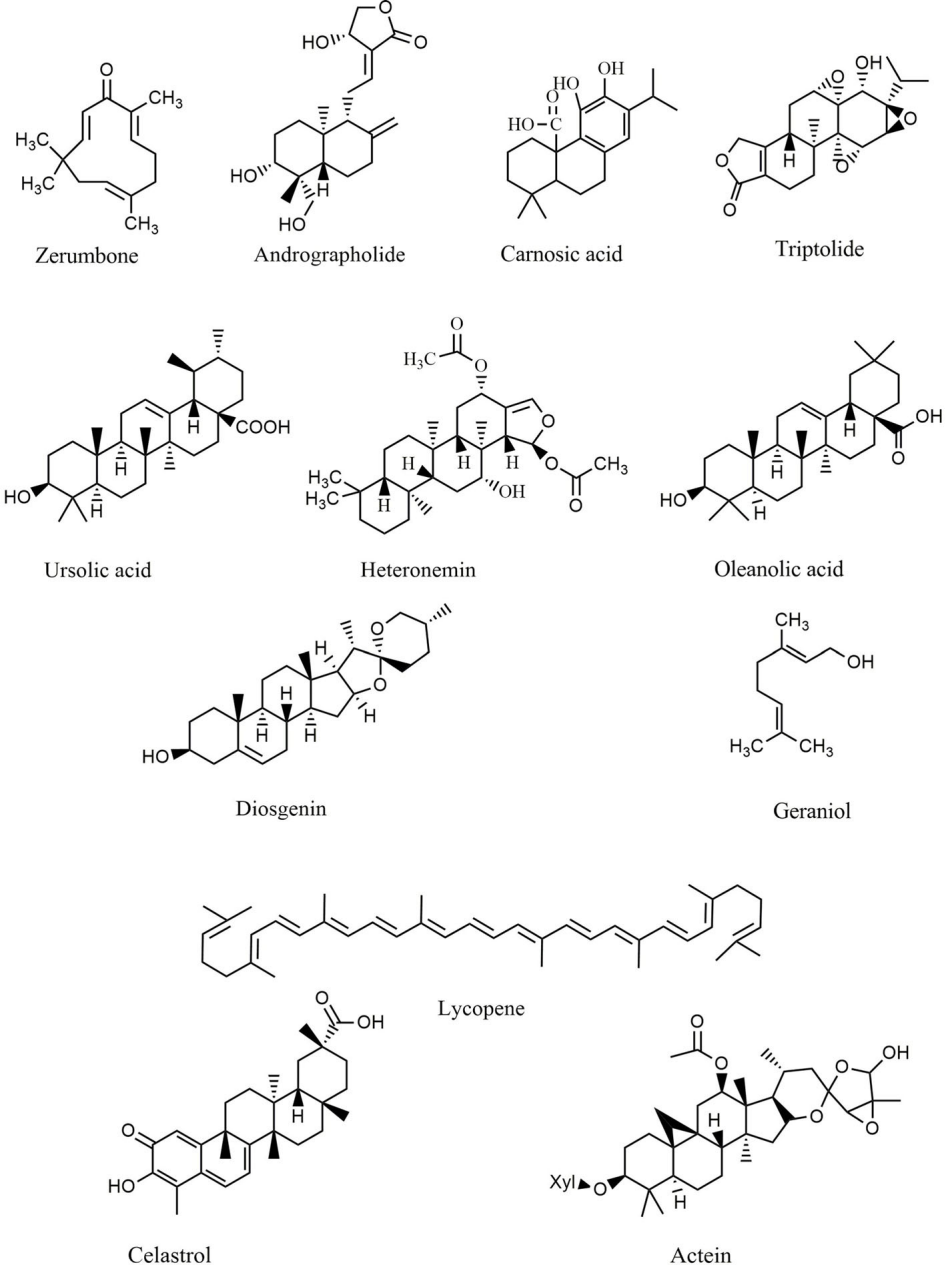
Chemical structures of selected terpenes/terpenoids that modulate the TLR/NF-κB/NLRP signaling in cancer.

Terpenes and terpenoids represent large classes of natural products isolated from multiple vegetative sources. These phytochemicals exert several therapeutic effects, including anticancer, cardioprotective, neuroprotective, and hepatoprotective activities. Each terpene/terpenoid compound is composed of several isoprenes (a five-carbon unit) that are assembled in thousands of ways. Zerumbone (**Figure 5**), an important sesquiterpene isolated from ginger, suppressed lung and colon tumors in mice *via* induction of apoptosis and inhibition of proliferation, heme oxygenase 1 (HO-1), and NF-κB expression (216). Andrographolide (**Figure 5**) is a bioactive phytochemical obtained from *Andrographis paniculata* that belongs to the diterpenoid compounds. Likewise, andrographolide showed antitumor activity against B16 melanoma cells, C57BL/6J mice (217), and RIP1-Tag2 mice (218) *via* suppression of TLR4/NF-κB signaling, thereby reducing the expression of CXCR4 and Bcl-6. Additionally, treatment with andrographolide inhibited the proliferation of SW620 colon cancer cells *in vitro via* interfering with the TLR4/NF-κB/MMP-9 signaling pathway (219).

Carnosic acid (**Figure 5**), one of the principal phenolic diterpenes isolated from rosmarinus officinalis, possesses antimicrobial, antioxidative, and anti-carcinogenic properties. Carnosic acid nanoparticles induced apoptosis in Bel7402 and MHCC97-H hepatic carcinoma cell lines *in vitro via* suppression of NF-κB, caspase-3, TLR4, MyD88, TRAF-6, interleukin 1 receptor associated kinase 1 (IRAK-1), and IRAK-4 (220). Similarly, triptolide (**Figure 5**) is an active natural phytochemical isolated from *Tripterygium wilfordii* Hook F that exhibits a wide range of pharmacological effects, including anti-diabetic, neuroprotective, anti-inflammatory, and antitumor activities. Triptolide is a well-known diterpenoid that blocks the NF-κB survival pathways and activates ERK1/2 and p38a in PC3 cancer cells (221). Additionally, triptolide decreased the expression of MMP-9, AP-1, and NF-κB signaling pathways in MCF-7 cells (222) and attenuated the angiogenesis and invasion of thyroid carcinoma cells *in vitro* and *in vivo* (223–226). Ursolic acid (**Figure 5**) exerts anti-inflammatory activity *via* suppression of the TLR4-MyD88 pathway and decreased production of inflammatory factors, including IL-1β, TNF-α, and IL-6 in abelson murine leukemia macrophage (RAW 264.7) cells (227). In a similar study, ursolic acid demonstrated significant *in vitro* and *in vivo* anticancer activity against DU145 and LNCaP prostate adenocarcinoma cells, as well as a mouse model *via* diminished CXCR4/CXCL-12 signaling axis and reduced activation of NF-κB (228). Furthermore, ursolic acid induced apoptosis and inhibited growth in several *in vitro* pancreatic and colon cancer models by interfering with PI3K/Akt/NF-κB, STAT, GSK, TRAIL, and JNK pathways (229, 230). In addition to ursolic acid, heteronemin (**Figure 5**) showed significant antiproliferative effects against AML cells *via* targeting NF-κB, Ras, MAPK, AP-1, and c-myc (231). Soyasaponins, bioactive phytochemicals found in a multitude of legumes, downregulated TLR4/MyD88 signaling and decreased TNF-α, IL-6, COX-2, NO, IL-1β, and iNOS in inflammatory macrophages (232). Oleanolic acid (**Figure 5**), another triterpenoid structure, and its synthetic derivative SZC014 showed considerable antitumor effects against the hepatocellular cancer cell lines HepG2, interfering with NF-κB and nuclear factor-erythroid factor 2-related factor 2 (Nrf-2)/antioxidant response element (ARE) signaling (233, 234). Additionally, inhibition of IκB kinase and suppression of NF-κB signaling are the primary anticancer mechanisms of lycopene (**Figure 5**) in breast and prostate cancer cells (235).

The monoterpene geraniol (**Figure 5**) is an acyclic isoprenoid derived from essential oils. Geraniol attenuated tongue carcinogenesis *via* decreased activation of NF-κB (236). Additionally, the NF-κB and STAT3 pathways are the chemopreventive mechanisms of andrographolide *via* inhibition of inflammatory mediators (237). Moreover, treatment with celastrol (**Figure 5**) downregulated NF-κB and reduced the expression of IL-6 in prostate and breast cancer cells (238, 239). In a similar study, actein (**Figure 5**) strongly suppressed the growth of MDA-MB-453 cells by enhancing the cytoplasmic calcium and modulating the MAPK/ERK kinase (MEK) and NF-κB pathways (240).

Diosgenin (**Figure 5**), a known steroidal triterpenoid with two pentacyclic rings, is found in *Trigonella foenum graecum* (241). Progesterone, pregnenolone, cortisone, and other steroids can be synthesized from diosgenin, which comprises more than 60% of commercial synthetic steroids (242). Diosgenin has shown several biological activities, including anticancer, antidiabetic, anti-infectious, anti-inflammatory, and anticoagulant effects (242). Diosgenin exerted significant antitumor potential *via* induction of apoptosis and suppression of inflammation. It also inhibited the invasion, metastasis, angiogenesis, and proliferation of various cancer cell lines. Accordingly, targeting inflammation-related pathways, including NF-κB and STAT3, is one of the main anticarcinogenic mechanisms of diosgenin (241, 242). Diosgenin displayed antiproliferative effects in HEp-2 and M4Beu cell lines *via* enhancing the production and release of apoptosis-inducing factors, increasing the Bax/Bcl-2 ratio, modulating caspase-3, and facilitating the activation of p53 (243). Furthermore, diosgenin induced apoptosis in the colon cancer cell lines HT-29 and HCT-116 *in vitro* by interfering with COX-2 signaling and increasing DNA fragmentation, caspase-3, and 5-lipoxygenase activity (244). Additionally, diosgenin sensitized colorectal cancer cells to apoptosis induced by TRAIL *via* activation of the p38MAPK pathway, overexpression of death receptor-5 (DR5), and downregulation of the Akt pathway (245).

Several compounds belonging to the terpenes and terpenoids classes exhibit antineoplastic properties by affecting various stages of tumor development, including suppression of the initiation and progression of tumorigenesis through promoting apoptosis, cell cycle arrest, inhibition of metastasis, angiogenesis, invasion, and downregulation of several intracellular signaling pathways, including TLR4, STAT3, NF-κB, and MMP-9. These compounds are promising therapeutic agents due to the massive progression in delineating the details of their anticancer action. **Table 2** provides the various anticancer terpenes/terpenoids that interfere with the TLR/NF-κB/NLRP pathway to counter chemoresistance.

**Table 2:** Anticancer terpenes/terpenoids interfering with the TLR/NF-κB/NLRP pathway and cross-linked mediators against chemoresistance.

Compound	Types of study	Cell line(s)/tumor model(s)	Mechanisms of action	References
Zerumbone	*In vivo*	Mouse model of colorectal and lung caner	↓NF-κB; ↑Apoptosis; ↓proliferation; ↓HO-1	(216)
Andrographolide	*In vitro* and *in vivo*	Murine tumor cell (B16 melanoma); C57BL/6J mice	↓TLR4/NF-κB signaling; ↓CXCR4; ↓Bcl-6	(217)
	*In vitro* and *in vivo*	RIP1-Tag2 mice models; Insulinoma cell (β-TC-6)	↓TLR4/NF-κB signaling	(218)
	*In vitro*	Human colon cancer cell (SW620)	↓TLR4/NF−κB/MMP−9	(219)
Carnosic acid	*In vitro* and *in vivo*	Xenograft mice model; BALB/c Akt-knockout mice; Liver cancer cell (MHCC97-H and Bel7402)	↓NF-κB; ↓TLR4; ↑caspase-3; ↓MyD88; ↓TRAF-6; ↓IRAK-1; ↓IRAK-4	(220)
Triptolide	*In vitro* and *in vivo*	FEN1 E160D mice; Prostate cancer cell (PC3)	↓NF-κB; ↑ERK1/2; ↑p38	(221)
	*In vitro*	Breast cancer cell (MCF-7)	↓NF-κB; ↓MMP-9; ↓AP-1	(222)
	*In vitro* and *in vivo*	Anaplastic thyroid carcinoma cells (TA-K and 8505C); Nude mice	↓NF-κB; ↓angiogenesis; ↓cell invasion; ↓cyclin D1; ↓VEGF	(224)
	*In vitro*	Hepatocellular carcinoma cell (MHCC-97H)	↓NF-κB; ↓MMP-9; ↓invasion; ↓tumorigenesis	(225)
	*In vitro*	Gastric adenocarcinoma (AGS)	↓NF-κB signaling	(223)
	*In vitro* and *in vivo*	Gastric tumor cell (MGC-803 and HGC-27); Xenograft mice	↓Notch1; ↓NF-κB; ↓RBPJ, ↓IKKα, IKKβ	(226)
Ursolic acid	*In vitro*	Abelson murine leukemia macrophage (RAW 264.7)	↓TLR4-MyD88 pathway; ↓IL-1β; ↓TNF-α; ↓IL-6	(227)
	*In vitro* and *in vivo*	Prostate cancer cells (DU145, LNCaP); Transgenic mouse model of prostate adenocarcinoma (TRAMP mice)	↓NF-κB; ↓CXCR4; ↓CXCR4/CXCL-12 signaling	(228)
	*In vitro*	Colon cancer cell (SW480 and LoVo)	↑Apoptosis; ↓MMP-9; ↓COX-2; ↓p300/NF-κB signaling	(229)
	*In vitro*	Breast cancer cell (T47D, MCF-7, MDA-MB-231	↓NF−κB; ↓viability; ↑apoptosis; ↓cyclin−D1; ↑caspase−3	(230)
Heteronemin	*In vitro*	Acute myeloid leukemia cell (HL-60)	↓NF-κB; ↓Ras; ↓MAPK; ↓AP-1; ↓c-myc	(231)
Soyasaponin A1, A	*In vitro*	Abelson murine leukemia virus-induced tumor macrophage (RAW 264.7)	↓TLR4/MyD88 signaling	(232)
Oleanolic Acid	*In vitro*	Hepatocellular cancer cells (HepG2)	↓NF-κB; ↓Nrf-2/ARE	(233)
	*In vitro* and *in-silico*	Hepatocellular cancer cells (HepG2)	↓NF-κB; ↓Nrf-2	(234)
Lycopene	*In vitro*	Breast cancer cell (MDA-MB-231); Prostate cancer cell (PC3)	↓NF-κB; ↓IκB kinase; ↓IKKβ kinase	(235)
Geraniol	*In vivo*	Wistar albino rats	↓NF-κB	(236)
Andrographolide	*In vitro*	Abelson murine leukemia virus-induced tumor macrophage (RAW 264.7)	↓NF-κB; ↓STAT3; ↓iNOS; ↓COX-2	(237)
Celastrol	*In vitro*	Prostate carcinoma cell (PC-3)	↓NF-κB; ↓IL-6	(238)
	*In vitro*	Breast cancer cells (MDA-MB-468, MDA-MB-231)	↓NF-κB; ↓cell migration; ↓cell invasion; ↓IL-6	(239)
Actein	*In vitro*	Breast cancer cells (MDA-MB-453)	↓NF-κB; ↓MEK; ↑cytoplasmic calcium	(240)
Diosgenin	*In vitro*	Laryngocarcinoma cells (HEp-2); Human melanoma cells (M4Beu)	↑Apoptosis; ↑AIF; ↑Bax/Bcl-2 ratio; ↑ p53	(243)
	*In vitro*	Human colorectal cancer cells (HT-29, HCT-116)	↑Apoptosis; ↑COX-2; ↑DNA fragmentation; ↑caspase-3; ↑5-lipoxygenase	(244)
	*In vitro*	Human colorectal cancer cells (HT-29)	↑Apoptosis; ↑p38 MAPK; ↑DR5; ↓Akt	(245)

### Alkaloids

**Figure 6:**
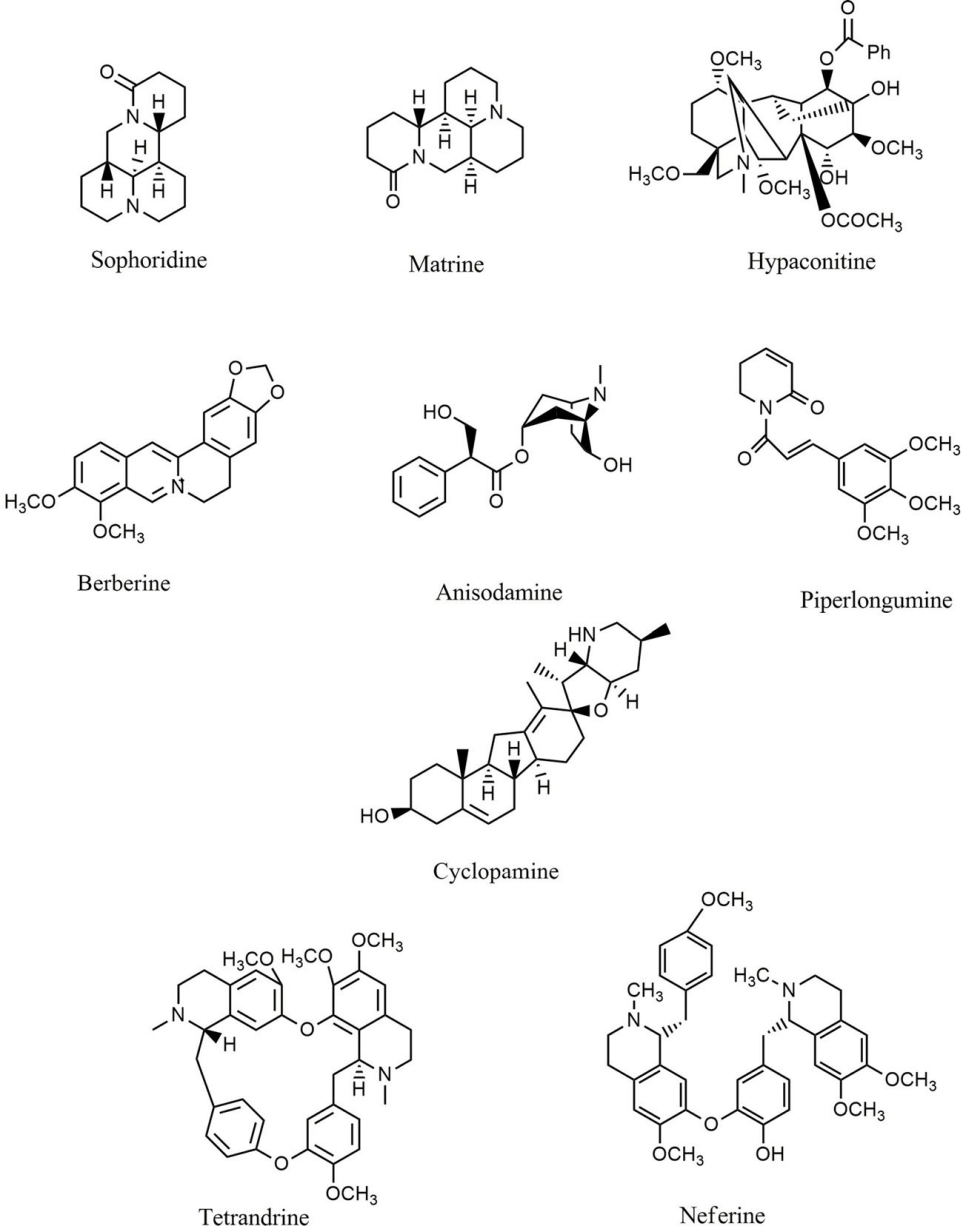
Chemical structures of selected alkaloids that modulate the TLR/NF-κB/NLRP signaling in cancer.

Alkaloids are another group of plant secondary metabolites that hold a substantial role in the defensive and internal immune processes of plants. Antioxidative, antimicrobial, antiprotozoal, anti-inflammatory, and anticancer activities are some of the important biological activities of alkaloids. Vinblastine and camptothecin are two important compounds belonging to the alkaloid group that have been properly developed and received the Food and Drug Administration’s approval for the treatment of different cancers. Sophoridine (**Figure 6**) inhibited macrophage-mediated immunosuppression by interfering with the TLR4/IRF-3 pathway, downregulating IL-10, CD206, and arginase 1 (Arg-1), and upregulating IL-12α, IFN-β, and iNOS in RAW264.7 and MFC cell lines (246). Moreover, matrine (**Figure 6**) demonstrated anticancer activity by regulating immunity, increasing TLR8 and TLR7, and activating MyD88-dependent signaling (247). Additionally, matrine inhibited the invasion and proliferation of breast and prostate cancer cells via downregulation of VEGF/Akt, MMP-2, and MMP-9 through the NF-κB signaling pathways (248, 249). Furthermore, hypaconitine **(** **Figure 6**), another alkaloidal agent, inhibited the adhesion, invasion, and migration of the A549 cell line (250). In addition to hypaconitine, alpinetin showed significant anticancer properties, diminishing the transcription of HIF-1α, NF-κB, and the ROS/NF-κB/HIF-1α axis in breast cancer cells (251). In a similar study, berberine (**Figure 6**), a well-known alkaloid, inhibited the proliferation of lung cancer cells and induced apoptosis through upregulation of Bcl-2/Bax, NF-κB, COX-2, MMP-2, and Akt/ERK pathways (252–254). Likewise, berberine suppressed the NLRP3 inflammasome in MDA-MB-231 cells *in vitro* (255). Treatment with berberine inhibited MMP-2, MMP-9, NF-κB, focal adhesion kinase (FAK), urokinase-type plasminogen activator (u-PA), and IKK in SCC-4 cancer cells (256). Additionally, berberine prevented DMBA-induced breast carcinogenesis in Sprague Dawley rats (257) and inhibited the growth of MDA-MB-231 cells *via* decreased IL-6, TNF-α, and NF-κB (258). Furthermore, berberine exerted anticancer activity *via* targeting variant pathways, such as NF-κB/COX-2, p38/JNK, AP-2/telomerase reverse transcriptase (hTERT), cytochrome-c/caspase, and HIF-1α/VEGF signaling in human gastric and NSCLC cell lines (259, 260). The alkaloid anisodamine (**Figure 6**) is another anticancer compound that inhibited the growth, invasion, and proliferation of HepG2 cells and suppressed the expression and activation of IFN-γ, IL-27, NLRP3, IL-4, and TNF-α (261). In a similar study, a steroidal alkaloid, cyclopamine (**Figure 6**), induced apoptosis and suppressed the proliferation of HEL and TF1a cells *via* induction of PKC, COX-2 overexpression, PARP cleavage, and modulation of MAPK/Akt signaling (262). The main anticancer mechanisms of cepharanthine and tetrandrine (**Figure 6**) against Jurkat T leukemia cells (263) are the modulation of PI3K/Akt/mTOR signaling, induction of apoptosis, cell cycle arrest, and phosphorylation of JNK and p38. Another alkaloid structure, piperlongumine (**Figure 6**), appears to have anticancer properties *via* downregulating c-Met expression and NF-κB activity in renal, colon, lung, and prostate carcinoma cells (264–268). Harmine (269), fangchinoline (270), sinapine (271), gramine (272), cepharanthine (273), piperine (274, 275), lamellarin D (276), ipobscurine (277), chelerythrine (278), dihydrochelerythrine (279), tryptanthrin (280), and neferine (**Figure 6**) (281) are some of the other alkaloid agents that exert significant anticancer activity through modulation of VEGF, AP-1, fibroblast growth factor receptor 4 (FGFR4)/fibroblast growth factor receptor substrate 2α (FRS2α)-ERK1/2, NF-κB, Nrf-2/Kelch-like ECH-associated protein 1 (Keap-1), MMP-2, MMP-9, and STAT3.

Overall, alkaloids are phytochemicals with significant potential to suppress the *in vitro* and *in vivo* growth/invasion of various cancers. By interfering with TLR/NF-κB/NLRP, alkaloids, especially berberine, matrine, and evodiamine, diminish cancer chemoresistance and facilitate the induction of apoptosis, inflammation, oxidative stress, and autophagy in cancer cells. **Table 3** provides various anticancer alkaloids that interfere with the TLR/NF-κB/NLRP pathway against chemoresistance.

**Table 3:** Anticancer alkaloids interfering with the TLR/NF-κB/NLRP pathway and interconnected mediators against chemoresistance.

Compound	Types of study	Cell line(s)/tumor model(s)	Mechanisms of action	References
Sophoridine	*In vitro*	Mouse gastric carcinoma cell (MFC)	↓TLR4/IRF-3 pathway; ↓IL-10; ↓CD206; ↓Arg-1; ↑IL-12α; ↑IFN-β; ↑INOS	(246)
Matrine	*In vitro*	Mouse lung cancer cells	↑TLR7; ↑TLR8; ↑MyD88; ↑TRAF-6; ↑IKK; ↑IL-12; ↑IL-6; ↑TNF-α	(247)
	*In vitro*	Breast cancer cell (MDA-MB-231)	↓NF-κB; ↓ratios of Bcl-2/Bax; ↓p-Akt; ↓MMP-9; ↓MMP-2; ↓EGF; ↓VEGFR1	(248)
	*In vitro* and *in vivo*	Prostate cancer cells (DU145 and PC3); Xenograft Balb/c nude mice model	↓NF-κB; ↓MMP-9; ↓MMP-2; ↓p-p65	(249)
Hypaconitine	*In vitro*	Human lung carcinoma cell (A549)	↓Cell adhesion; ↓cell invasion; ↓Cell migration; ↓TGF-β1; ↓NF-κB	(250)
Alpinetin	*In vitro* and *in vivo*	Breast cancer cells (MCF-7,4T1, MDA-MB-231); BALB/C female mice	↓NF-κB; ↓HIF-1α transcription; ↓ROS/NF-κB/HIF-1α	(251)
Berberine	*In vitro*	Human lung carcinoma cell (A549)	↓JAK2/VEGF/NF-κB/AP-1; ↓cell proliferation; ↑apoptosis; ↓MMP-2; ↓Bcl-2/Bax	(253)
	*In vitro*	Human lung cells (H1299 and A549)	↓NF-κB/COX-2; ↓AP-2β/hTERT; ↓Akt/ERK; ↑caspase/cyt c signaling	(252)
	*In vitro*	Human lung cell (A549)	↑Apoptosis; ↓cyclins	(254)
	*In vitro*	Breast cancer cell (MDA-MB-231)	↓NLRP3; ↓IL-1β; ↓IL-1α; ↓P2X7; ↓IL-6; ↓TNF-α	(255)
	*In vitro*	Tongue SCC cells (SCC-4)	↓NF-κB; ↓cell migration; ↓invasion; ↓FAK; ↓ IKK; ↓ MMP-2; ↓ MMP-9; ↓ u-PA	(256)
	*In vivo*	Sprague Dawley rats	↓NF-κB; ↓PCNA; ↓malonaldehyde; ↓IL-1β; ↓IL-6; ↓TNF-α; ↓SOD; ↓CAT; ↓GSH	(257)
	*In vitro*	Breast cancer cell (MDA-MB-231)	↓NF-κB; ↓TNF-α; ↓IL-6	(258)
	*In vitro*	Human gastric cancer cell (SNU-1)	↓NF-κB; ↓p38/JNK pathway; ↑Apoptosis; ↑Caspase	(260)
	*In vitro*	Human non-small-cell lung cancer cell (NSCLC)	↓p50/p65 NF-κB; ↓AP-2α; ↓AP-2β; ↓pAkt; ↓pERK; ↑cyt c release; ↑cleavage of caspase; ↑cleavage PARP	(259)
Anisodamine	*In vitro* and *in vivo*	Hepatocellular carcinoma cells (HepG2); BALB/C nude mice	↓NLRP3; ↓IFN-γ; ↓IL-27; ↓IL-4; ↓TNF-α	(261)
Cyclopamine	*In vitro*	Human erythroleukemia cells (HEL and TF1a)	↓Cell proliferation; ↑apoptosis; ↑COX-2; ↑PKC; ↑ PARP cleavage; ↓MAPK; ↓Akt	(262)
Cepharanthine & Tetrandrine	*In vitro*	T cell leukemia (Jurkat)	↑Apoptosis; ↑p-JNK; ↑Phosphorylation of p38; ↑cyclin A2; ↑cyclin B1; ↓cylcin D1; ⟂S phase cell cycle	(263)
Piperlongumine	*In vitro*	Prostate cancer cells (PC-3, LNCaP, DU-145)	↓NF-κB; ↓IL-6; ↓IL-8; ↓MMP-9; ↓ICAM-1	(264)
	*In vitro*	Renal cell carcinoma (PNX0010, 786-O)	↓NF-κB; ↓C-Met; ↓Akt/mTOR; ↓Erk/MAPK; ↓STAT3	(266)
	*In vivo*	Mouse model of colon cancer	↓NF-κB; ↓COX-2; ↓JAK/STAT	(268)
	*In vitro*	Human lung cancer cell (A549)	↓NF-κB p65; ↓Akt; ↓Cyclin D1; ↓CDK4; ↓CDK6; ↓p-Rb; ↑pERK1/2	(267)
	*In vitro*	Multiple myeloma (U266); Breast cell cancer (MCF-7); T cell leukemia Jurkat; Lung adenocarcinoma cell (H1299); Squamous cell (SCC4); CML (KBM-5)	↓NF-κB; ↓cyclin D1; ↓Bcl-2; ↓VEGF; ↓Bcl-XL; ↓c-IAP-2; ↓ICAM-1; ↓survivin; ↓COX-2; ↓IL-6; ↓CXCR-4; ↓c-IAP-1; ↓c-Myc	(265)
Harmine	*In vitro* and *in vivo*	Melanoma cell (B16F-10); C57BL/6 mice	↓VEGF; ↓MMP; ↓TIMP; ↓iNOS; ↓COX-2	(269)
Fangchinoline	*In vitro*	Human CML cell (KBM5); Multiple myeloma cell (U266)	↓NF-κB; ↑apoptosis; ↓AP-1	(270)
Sinapine	*In vitro*	Breast cancer cell (MCF-7)	↓NF-κB; ↓FGFR4/FRS2α-ERK1/2	(271)
Gramine	*In vivo*	Male golden Syrian hamsters	↓NF-κB; ↓cell proliferation; ↓STAT3; ↓ EGFR/PI3K/Akt/mTOR; ↓JAK/STAT3	(272)
Cepharanthine	*In vitro* and *in vivo*	Human OSCC cells (B88 and HSC3); Athymic nude mice	↓NF-κB; ↓angiogenesis; ↓VEGF; ↓IL-8	(273)
Piperine	*In vivo*	Wistar rats’ models of colon cancer	↓NF-κB/Nrf-2/Keap-1/HO-1	(275)
	*In vitro* and *in vivo*	Human cervical cancer cell (HeLa); Mice xenograft models	↓STAT3/NF-κB; ↓Bcl-2; ↓p-STAT3	(274)
Lamellarin D	*In vitro*	Human leukemia cell (K562)	↑Apoptosis; ⟂G0/G1 cell cycle arrest; ↓CDK1; ↓smad3-5; ↓TGF-β; ↓IL-1β; ↓IL-6; ↓IL-8; ↑p27; ↑p53; ↑STGC3	(276)
Ipobscurine	*In vitro*	Melanoma cell (B16F-10);	↓NF-κB; ↑Caspase-3; ↑p53; ↑Bax; ↓Bcl-2; ⟂G1 cell cycle arrest	(277)
Chelerythrine	*In vitro*	Prostate cancer cells (DU145, PC-3)	↓MMP-2; ↓MMP-9; ↓uPA; ↓NF-κB; ↓AP-1; ↓p-p65, c-Fos; ↓c-Jun protein	(278)
Dihydrochelerythrine	*In vitro*	Human glioblastoma cells (U251 and GL-15); Murine glioblastoma cell (C6)	↓NF-κB; ↓cell viability	(279)
Tryptanthrin	*In vitro* and *in vivo*	Murine breast cancer model (4T1) Breast cancer cell (MCF-7)	↓NF-κB; ↑E-cadherin; ↓MMP-2; ↓Snail; ↓NOS1; ↓COX-2; ↓IL-2; ↓IL-10; ↓TNF-α	(280)
Neferine	*In vivo*	Wistar rats	↓NF-κB; ↓PI3K/AKT/mTOR	(281)

### Sulfur-Containing Compounds and Miscellaneous Agents

**Figure 7:**
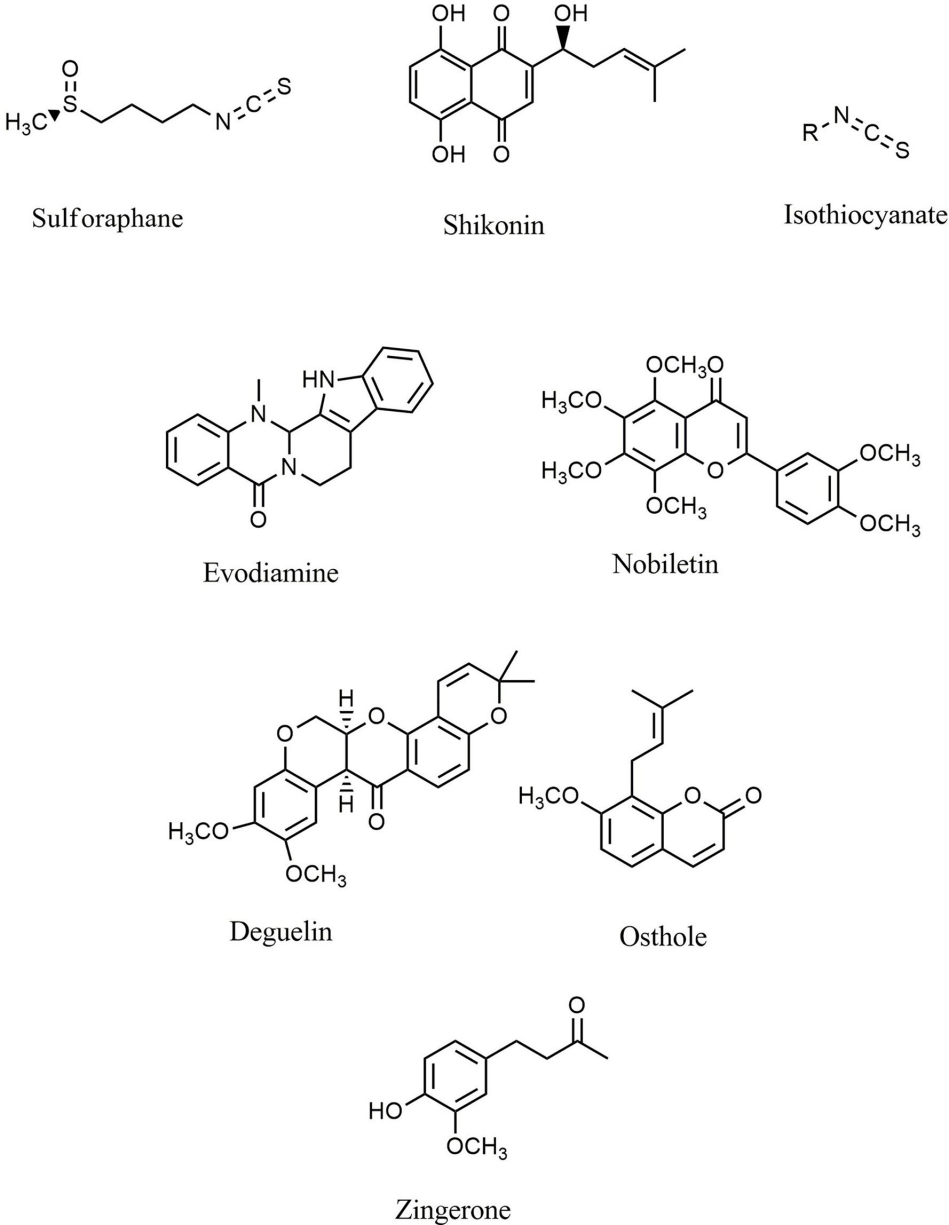
Chemical structures of selected sulfur-containing compounds and miscellaneous agents with effect on TLR/NF-κB/NLRP signaling in cancer.

Sulforaphane (**Figure 7**) suppressed TLR3-mediated NF-κB in PCI15A SCC cells (282). In addition, sulforaphane inhibited the expression of MMP-9, phosphorylation of IκB, and activation of NF-κB in MCF-7 cells (283). Another sulfur-containing compound, shikonin (**Figure 7**), appears to have anticancer properties *via* suppressing the migration, adhesion, viability, and invasion of gastric (MGC-803) and hepatocellular (Huh7and BEL7402) cancer cells *in vitro via* the TLR2/NF-κB and receptor-interacting protein 1 (RIP1)/NF-κB pathways (284, 285). In a similar study, phenethyl isothiocyanate (**Figure 7**) in combination with xanthohumol activated Nrf-2 and suppressed NF-κB in pancreatic cancer cells (286) and B-cell acute lymphocytic leukemia (287). Moreover, inhibition of NF-κB and MAPK signaling is the main anticancer mechanism of phenethyl isothiocyanate against AGS cell lines (288). The indolequinazoline alkaloid evodiamine is widely present in many medicinal plants belonging to the tetradium family. Evodiamine (**Figure 7**) and its derivatives targeted the c-Met, NF-κB, Smad2/3, and TGF-β/hepatocyte growth factor pathways in prostate, hepatocellular, lung, and melanoma carcinoma cells (289–292). It was reported that microsclerodermin A inhibited NF-κB, promoted apoptosis, and diminished cytokine release in pancreatic and breast cancer cells (293, 294). Additionally, treatment of prostate, lung, and pancreatic cancer cells (295–298) with matrine and oxymatrine inhibited angiogenesis, VEGF, NF-κB, and CXCR4. Nobiletin (**Figure 7**) is a citrus flavonoid with several pharmacological activities, including anticarcinogenic, antioxidative, neuroprotective, and anti-inflammatory effects. Nobiletin modulated the activity of the Cd36/STAT3/NF-κB pathway and inhibited the growth and migration of breast cancer cell lines MCF-7 and MDA-MB-231 (299). Similarly, it leads to the downregulation of Akt, HIF-1α, NF-κB, and VEGF in OVCAR-3 ovarian cancer cells and suppression of TRIF/receptor interacting serine/threonine kinase 1 (RIPK1)/Fas associated *via* death domain (FADD), TRIF protein, caspase-8, and TLR3/IRF-3 in LNCaP and PC-3 cell lines (300, 301). Additionally, nobiletin diminished the invasion and migration of AGS cells and downregulated FAK/PI3K/Akt, c-Raf, Rac-1, cell division control protein 42 homolog (Cdc42), as well as the NF-κB, MMP-2, and MMP-9 signaling pathways (302).

*Ulmus davidiana* Nakai glycoprotein (303), phenylpropenone derivatives (304), libanoridin (305), and alisol B 23-acetate (306) decreased the proliferation and migration of colon carcinoma cells *via* inactivation of inflammatory and angiogenic pathways. Deguelin (**Figure 7**) downregulated EGFR, c-Myc, pAkt, p-ERK, p-STAT3, c-met, survivin, and NF-κB in xenograft athymic mice models of breast cancer cell lines MDA-MB-231, MDA-MB-468, BT-549, and BT-20 (307). Withaferin A, a steroidal lactone presents in *Withania somnifera*, exerts several pharmacological activities such as anti-inflammatory, anticancer, and cardioprotective effects. Withaferin A significantly downregulated the liver X receptor-a, NF-κB, and angiogenesis pathways in the hepatocellular carcinoma cell line QGY-7703 (308). Furthermore, withaferin A downregulated caspase-1 and AIM-2 in THP-1 cells (309). Similarly, Zingerone (vanillylacetone) (**Figure 7**) is another known agent with anticancer properties that inhibited NF-κB, p42/44, and MAPK/AP1signaling and attenuated the migration and invasion of human hepatocellular carcinoma cells (310). Moreover, osthole and ophiopogonin D (**Figure 7**) suppressed the PI3K/Akt, NF-κB, and AP-1 pathways in A549 and H1299 lung cancer cells (311–314). Embelin (315), plumbagin (316, 317), indole glucosinolates (318), thymoquinone (319, 320), decursinol angelate (321), polysaccharide agaricus blazei murill (322), and 19-a-hydroxyurs-12(13)-ene-28-oic acid-3-O-b-D-glucopyranoside (HEG) (323) are some of the other miscellaneous agents that have promising anticancer potential against MDAMB-231, pancreatic PANC1, Ehrlich ascites carcinoma, fibrosarcoma HT1080, and chronic myeloid leukemia (CML) KBM-5 cancer cell lines *in vitro* and *in vivo*.

Overall, sulfur-containing compounds demonstrate critical biological properties and therefore have meaningful potential for the prevention and treatment of cancers. These phytochemicals could target multiple signals affecting cancer progression, especially TLR/NF-κB/NLRP signaling and cross-talked pathways, to support cancer immunotherapy and chemotherapy. **Table 4** provides the various anticancer sulfur and miscellaneous compounds in interfering with the TLR/NF-κB/NLRP pathway against chemoresistance.

**Table 4:** Anticancer sulfur compounds and miscellaneous agents interfering with the TLR/NF-κB/NLRP pathway and interconnected mediators against chemoresistance.

Compound	Types of study	Cell line(s)/tumor model(s)	Mechanisms of action	References
Sulforaphane	*In vitro*	Squamous cell carcinoma (PCI15A)	↓TLR3; ↓NF-κB	(282)
	*In vitro*	Breast cancer cell (MCF-7)	↓NF-κB; ↓MMP-9; ↓Phosphorylation of IκB;	(283)
Shikonin	*In vitro*	Human gastric cancer cell (MGC-803)	↓TLR2; ↓NF-κB; ↓Cell invasion; ↓MMP-2; ↓MMP-7; ↓p65 NF-κB	(285)
	*In vitro* and *in vivo*	Hepatocellular carcinoma (Huh7 and BEL7402); Xenograft mice	↓RIP1/NF-κB; ↓Akt	(284)
Xanthohumol & phenethyl isothiocyanate	*In vitro*	Pancreatic cancer cell (PANC-1)	↓NF-κB; ↑Nrf-2; ↑GSTP; ↑NQO1; ↑SOD; ↓MMP-2; ↓MMP-7; ↓MMP-9; ↓FAK	(286)
Xanthohumol	*In vitro* and *in vivo*	B-cell acute lymphocytic leukemia; Xenograft mouse model	↓NF-κB; ↓FAK; ↓Akt	(287)
Phenethyl isothiocyanate	*In vitro*	Gastric cancer cell (AGS)	↓Cell migration; ↓cell invasion; ↓MAPK; ↓NF-κB	(288)
Evodiamine	*In vitro*	Prostate cancer (DU145, PC-3)	↓Cellular growth; ↑apoptosis	(291)
	*In vitro*	Lung cancer cell line (A549)	↓Akt/NF−κB; ↑apoptosis; ↓GSH; ↓SHH/GLI1	(290)
	*In vitro*	Melanoma cell (A375-S2)	↓PI3K/Akt/caspase; ↓Fas-L/NF-κB	(289)
Evodiamine derivatives	*In vitro* and *in vivo*	Hepatocellular carcinoma cells (HepG2, Huh-7, MHCC-LM9); Nude male mice	↓Topo I; ⟂G2/M cell cycle arrest; ↑Apoptosis; ↓cell migration; ↓cell invasion; ↓tumor volume; ↓tumor weight	(292)
Marine	*In vitro*	Breast cancer cell (MDA-MB-231); Monocytic leukemia cell (THP-1)	↓NF-κB; ↓cytokine release; ↓phosphorylation of p65; ↓phosphorylation of IκB	(294)
	*In vitro*	Pancreatic cancer cells (PANC-1, AsPC-1, MIA PaCa-2, BxPC-3)	↓NF-κB; ↑Apoptosis	(293)
Matrine	*In vitro*	Human lung carcinoma cell (A549); Pancreatic cancer cells (MIA PaCa-2); Prostate cancer cells (DU145)	↓NF-κB; ↓CXCR4; ↓MMP-9, MMP-2	(298)
	*In vitro*	Prostate cancer cell (PC-3, DU145)	↓NF-κB	(296)
	*In vitro*	Prostate cancer cell (DU145)	↓NF-κB; ↓GADD45B; ↓MMP-2; ↓MMP-9	(297)
Oxymatrine	*In vitro* and *in vivo*	Pancreatic cancer cells (PANC-1); BALB/C male mice	↓NF-κB; ↓VEGF	(295)
Nobiletin	*In vitro*	Breast cancer cells (MDA-MB-231, MCF-7)	↓Cd36/Stat3/NF-κB; ↓angiogenesis; ↓migration; ↓invasion; ↓STAT3; ↓CD36;	(299)
	*In vitro*	Ovarian cancer cells (OVCAR-3, A2780/CP70)	↓NF-κB; ↓tumor growth; ↓angiogenesis; ↓Akt; ↓HIF-1α; ↓VEGF	(300)
	*In vitro*	Prostate cancer cell (PC-3, LNCaP)	↓TRIF protein; ↓caspase-8; ↓TRIF/RIPK1/FADD; ↓TLR3/IRF-3	(301)
	*In vitro*	Gastric adenocarcinoma (AGS)	↓NF-κB; ↓FAK/PI3K/Akt	(302)
UDN glycoprotein	*In vivo*	Male mice (ICR)	↓NF-κB	(303)
Phenylpropenone derivatives	*In vitro*	Human colon carcinoma cell (HT-29)	↓NF-κB; ↓PERK; ↓RTKs; ↓VEGF	(304)
Libanoridin	*In vitro*	Human colon carcinoma cell (HT-29)	↓iNOS; ↓COX-2; ↓TNF-α; ↓IL-1β	(305)
Alisol B 23-acetate	*In vivo*	Male C57BL/6J mice	↓TLR; ↓NF-κB; ↓MAPK; ↓phosphorylation of p38; ↓PERK; ↓PJNK	(306)
Deguelin	*In vitro* and *in vivo*	Xenograft athymic mice; MDA-MB-231, MDA-MB-468, BT-549 and BT-20 cells	↓NF-κB; ↓EGFR; ↓c-Myc	(307)
Withaferin A	*In vitro*	Hepatocellular carcinoma cell (QGY-7703)	↓NF-κB; ↓liver X receptor-a; ↓angiogenesis	(308)
	*In vitro*	Monocytic leukemia cell (THP-1)	↓Caspase-1; ↓AIM-2; ↓TGF-β	(309)
Zingerone	*In vitro*	Hepatocellular carcinoma cell (SNU182)	↓Cell migration; ↓cell invasion; ↓MMP-2; MMP-9; ↓Smad2/3; ↓NF-κB; ↓P42/44 MAPK/AP1	(310)
Ophiopogonin D	*In vitro*	Lung cancer cell (A549)	↓NF-κB; ↓cell proliferation; ↓PI3K/Akt; ↓AP-1	(314)
Osthole	*In vitro*	Lung cancer cell (A549)	↓NF-κB	(312)
	*In vitro*	Lung adenocarcinoma cells (H1299 and A549)	↓NF-κB; ↓MMP-9	(311)
	*In vitro*	Cervical cancer cells (HeLa, SiHa, C−33A and CaSki)	↓ATM/NF−κB; ↑E−cadherin; ↓vimentin; ↑DNA damage; ↓NF-κB	(313)
Embelin	*In vitro* and *in vivo*	Breast cancer cell (MDA-MB-231)	↓NF-κB; ↓Cell invasion; ↓Cell migration; ↓CXCR4; ↓MMP-9; MMP-2	(315)
Plumbagin	*In vitro* and *in vivo*	Pancreatic cancer cells (PANC1, BxPC3); Xenograft SCID male mice	↓NF-κB; ↓EGFR; ↓STAT3	(316)
	*In vitro*	Gastric cancer cells (SGC-7901, MKN-28, AGS)	↓NF-κB	(317)
Ondole glucosinolates	*In vitro* and *in vivo*	Ehrlich ascites carcinoma cells; Albino mice	↓NF-κB; ↓IL-6; ↓IL-1β; ↓TNF-α; ↓NO	(318)
Thymoquinone	*In vitro*	Human myeloid cells (KBM-5)	↓NF-κB; ↑Apoptosis; ↓VEGF	(319)
	*In vitro*	Metastatic human (A375) and mouse (B16F10) melanoma cells	↓NLRP3; ↓NF-κB	(320)
Decursinol angelate	*In vitro*	Fibrosarcoma cell (HT1080); Breast cancer cell (MDA-MB-231)	↓NF-κB; ↓PI3K; ↓ERK; ↓β1-integrin; ↓MMP-9	(321)
Polysaccharide agaricus blazei murill	*In vivo*	TLR2-/- mice	↓TLR2; ↑IL-12, ↓Arg-1; ↑iNOS	(322)
HEG	*In vivo*	Swiss albino Wistar	↓NF-κB; ↓COX-2; ↓PGE2	(323)

## Phytochemicals in Combination With Anticancer Drugs Potentiate Chemotherapy and Immunotherapy

Despite the recent progress in designing, synthesizing, and introducing new pharmaceutical drugs, natural products and bioactive molecules isolated from plants exert undeniable roles in the treatment of various cancers. Currently, the most important treatment option for malignant tumors is chemotherapy, which may lead to numerous side effects and promote drug resistance in patients. The use of natural products for the treatment of cancer is not only economically efficient, but also exerts multiple prophylactic, protective, and therapeutic roles in the treatment process that may reduce the side effects of chemotherapy and radiotherapy and decrease drug resistance (6, 13, 324–326).

Numerous studies have investigated the *in vitro* and *in vivo* advantages of adding curcumin to various anticancer treatment regimens. Liposomal curcumin has sensitized mouse models of cervical cancer to paclitaxel treatment (327). Curcumin also facilitated the induction of cell death by paclitaxel in MCF7 and MDA-MB-234 cell lines (328, 329). It was reported that curcumin induced cell apoptosis and increased paclitaxel sensitivity in cervical cancer cells through interfering with NF-κB, p53, and caspase-3 signaling (330). In similar studies, curcumin significantly sensitized breast cancer cells to cyclophosphamide and paclitaxel through modulation of NF-κB, protein kinase C (PKC), histone deacetylase (HDAC), and telomerase (331). Combining curcumin with oxaliplatin reversed the acquired resistance in an *in vitro* model of colorectal cancer by interfering with the CXC-chemokine/NF-κB pathway (332). In addition, curcumin increased the chemosensitivity of several platinum-based drugs *via* arresting the cell cycle in the G2/M phase, inducing apoptosis, and downregulating NF-κB (333). Similarly, co-delivery of curcumin and dasatinib led to the enhanced antitumor activity of dasatinib against colon cancer cells *via* diminished insulin-like growth factor type 1 receptor, c-Src, and EGFR signaling (334). Moreover, curcumin worked synergistically with tamoxifen to suppress the growth of MCF-7/LCC9 and MCF-7/LCC2 cells in an *in vitro* model of breast cancer by facilitating cell cycle arrest and inactivating the Akt/mTOR, Src, and NF-κB pathways (335). Furthermore, curcumin sensitized MDA-MB-231 cells to retinoic acid (336) and enhanced the efficacy, as well as diminished the toxicity, of doxorubicin (337). The anticancer potential of sorafenib against Huh7 cells and an athymic mice model of hepatocellular carcinoma was enhanced when combined with curcumin as evident by decreased expression of MMP-9 and NF-κB/p65 (338). Another well-known polyphenolic compound, resveratrol, sensitized PC3 and DU145 prostate cancer cells *in vitro* to cisplatin-induced apoptosis *via* inhibition of COX-2 and NF-κB pathways (339). In a similar study, resveratrol significantly increased the efficacy of cisplatin in xenografted mice and MDA-MB-231 cancer cells *via* decreased activity of p-ERK, TGF-β1, Smad2, vimentin, p-Akt, NF-κB, p-PI3K, and p-JNK (340). Furthermore, resveratrol sensitized colorectal cancer cells to 5-fluorouracil (5-FU) by inducing apoptosis and downregulating NF-κB (341). Additionally, resveratrol improved the gemcitabine-induced apoptosis of PaCa cells *via* inhibition of NF-κB and diminished expression of cyclin D1, VEGF, intercellular adhesion molecule-1 (ICAM-1), COX-2, and MMP-9 (342). Apigenin is another polyphenolic substance that potentiated the antitumor activity of several antineoplastic agents, including paclitaxel (343), tamoxifen (344), gemcitabine (345, 346), doxorubicin (347), and cisplatin (348) against various *in vitro* and *in vivo* cancer models. Polyphenol quercetin is another anticancer compound that works synergistically with paclitaxel (349, 350), tamoxifen (351), cisplatin (352, 353), adriamycin (354), and gemcitabine (355) to suppress the growth of various models of cancer *via* enhanced ROS production, cell cycle arrest, ER stress, and apoptosis. Similarly, various studies have reported the advantages of adding EGCG to enhance the antitumor activity of sunitinib, irinotecan, doxorubicin, gemcitabine, and cisplatin against human lung (A549, H460, and H1975) (356, 357), colorectal (HCT116 and RKO) (358), bladder (SW780 and T24) (359), pancreatic (MIA PaCa-2 and Panc-1) (360), and ovarian (OVCAR3 and SKOV3) (361) cancer cells, respectively. The results emphasized that EGCG could potentate the antineoplastic activity of the aforementioned drugs by increasing the sensitivity of the cancer cells, thereby enhancing their antiproliferative activity, damaging DNA, interfering with the NF-κB/MDM2/p53 pathway, inhibiting Akt, and elevating copper transporter 1 (CTR1). Likewise, cotreatment of naringin with doxorubicin (362) and paclitaxel (363) amplified their anticancer activity against human esophageal and prostate cancer cells, respectively. Moreover, baicalin improved the chemosensitivity to cisplatin (364) and doxorubicin (365) in lung and breast cancer cells, respectively, by inducing cell cycle arrest, apoptosis, and DNA damage. Furthermore, arctigenin sensitized various cancer cell lines, including SW620, HepG2, H460, HeLa, SW480, and K562, to cisplatin treatment (366–368). Morin (369), chrysin (370), and pterostilbene (371) are some of the other polyphenolic compounds that increased the cytotoxicity of antineoplastic agents in several *in vitro* models of cancer.

In addition to polyphenols, terpenes also showed a significant ability to increase the sensitivity of different cancer cell lines to various drugs, such as 5-FU, gefitinib, cisplatin, doxorubicin, and gemcitabine. Combination therapy of cisplatin and paclitaxel with zerumbone, a sesquiterpene agent, enhanced ROS production and p53 expression, as well as inhibited the JAK2/STAT3 pathway in prostate and lung cancer cells (372, 373). It was reported that andrographolide augmented the doxorubicin-mediated antitumor activity in different cancer cells through the blockade of JAK/STAT3 signaling (374, 375) and its coadministration with gemcitabine promoted apoptosis and inhibited STAT3 in pancreatic cancer cells (376). Furthermore, treatment with andrographolide increased cisplatin-induced antineoplastic activity against lung cancer cells (377). Carnosic acid is another triterpenoid compound that, in combination with cisplatin and tamoxifen, promoted apoptosis in lung (378) and breast (379) cancer cells. In addition to carnosic acid, triptolide showed significant anticancer properties and amplified the *in vitro* and *in vivo* anticancer activity of various chemotherapeutic agents, including cisplatin (380–382), paclitaxel (383), hydroxycamptothecin (384, 385), gemcitabine (386), and doxorubicin (387), in several cancer types, including bladder (EJ, UMUC3, and T24R2), breast (MDA-MB-231, BT549, and MCF7), lung (A549), and gastric (SC-M1) cancer cell lines. Another triterpenoid compound, ursolic acid, potentiated the therapeutic effects of gemcitabine, oxaliplatin, cisplatin, and paclitaxel in human pancreatic and colorectal cancer cells by promoting apoptosis and inhibiting the inflammatory microenvironment and NF-κB p65 signaling (388–391). Moreover, treatment with oridonin overcame antibiotic resistance and augmented the antineoplastic effects of doxorubicin, cisplatin, and gemcitabine *via* increased expression of Bax, induction of apoptosis, downregulation of Bcl−2, and inhibition of MMP in the *in vivo* and *in vitro* models of lung, breast, pancreatic, and ovarian cancers (392–395). In a similar study, ginsenoside Rg3 enhanced the cytotoxicity of paclitaxel in breast cancer cells by regulating the expression of Bax/Bcl-2 and suppressing NF-κB signaling (396). Additionally, ginsenoside Rg3 amplified cisplatin therapy in the lung cancer cell lines H1299, SPC-A1, and A549 by inhibiting the NF-κB pathway (397, 398). Another study found that co-administration of ginsenoside Rg3 and gefitinib increased the cytotoxicity of gefitinib against lung cancer *in vitro* (399). The results demonstrated that the main mechanisms by which ginsenoside Ro enhances the anti-malignant effects of 5-FU occurs by accumulating DNA damage, inhibiting DNA repair, downregulating DNA replication, and delaying the degradation of checkpoint kinase 1 (CHEK1) (400). The treatment combination of docetaxel and ginsenoside Rg3 increased activation of the apoptotic pathway in colon and prostate cancer cells *via* suppression of NF-κB (401, 402). Lycopene, a carotenoid agent, improved cisplatin-induced apoptosis in HeLa cancer cells *via* inhibition of NF-κB activation (403).

Alkaloids, like other secondary metabolites, enhance the *in vitro* and *in vivo* anticancer activities of various drugs by elevating their antiproliferative effects and promoting apoptosis and cell cycle arrest. The combined effects of doxorubicin and berberine on lung (404) and breast (405, 406) cancer cells inhibited the STAT3, high mobility group box 1 (HMGB1)-TLR4 axis and downregulated the expression of Nanog and miRNA-21. Cisplatin and berberine combined therapy also inhibited cell growth, promoted apoptosis, created DNA breaks, and interfered with miR-93/PTEN/Akt signaling in MCF-7 and A2780 cells (407, 408). Moreover, berberine increased the chemotherapy potential of irinotecan against *in vitro* models of colon cancer through the suppression of NF-κB (409). Another alkaloid compound, piperlongumine, induced apoptosis and potentiated the anticarcinogenic activity of doxorubicin, paclitaxel, oxaliplatin, cisplatin, and gemcitabine *via* suppression of the JAK2/STAT3 pathway and induction of oxidative stress in breast, intestinal, gastric, HNSCC, colorectal, and pancreatic cancer cells (410–415). In a similar study, harmine, in combination with paclitaxel, suppressed the invasion and migration of SGC-7901 and MKN-45 gastric cancer cell lines *via* downregulation of MMP-9 and COX-2 (416, 417). In addition, harmine suppressed the proliferation of pancreatic cancer cells *in vitro* by enhancing the cytotoxicity of gemcitabine (418). Likewise, several studies have reported the advantages of combining matrine with irinotecan and cisplatin to augment their antitumor activity against human colorectal (HT29) (419), urothelial bladder (EJ and T24) (420), liver (HepG2) (421), and cervical (U14) (422) cancer cell lines. The results suggested that matrine significantly potentiated the antineoplastic effects of both irinotecan and cisplatin and increased the sensitivity of the aforementioned cancer cells to treatment through induction of apoptosis, facilitation of cell cycle arrest, and enhanced activity of topoisomerase I, ROS, β−catenin, Bax, caspase-3, caspase-7, and caspase-9. Treatment with sophoridine inhibited the growth of lung cancer cells by amplifying cisplatin sensitivity *via* activation of Hippo and p53 signaling (423).

Sulforaphane could significantly sensitize human breast, lung, colorectal, and bladder cancer cells to variant chemotherapeutic agents *via* downregulation of NF-κB, induction of cell cycle arrest, and reduction of cyclin A and p-Akt (424–427). Furthermore, sulforaphane increased the *in vitro* and *in vivo* antiproliferative activity of salinomycin in colorectal cancer cells *via* diminished signaling of the PI3K/Akt pathway (428). Moreover, shikonin reversed gemcitabine tolerance in a xenograft model of pancreatic cancer *via* modulation of the NF-κB signaling pathway (429). Treatment with shikonin potentiated the antitumor efficacy of gefitinib in lung cancer cells through suppression of the PKM2/STAT3/cyclin D1 pathway (430). Shikonin also increased the sensitization of paclitaxel against esophageal cancer cells by promoting apoptosis (431). Additionally, shikonin enhanced 4-hydroxytamoxifen-induced apoptosis in breast cancer cells by activating mechanisms involved in apoptosis and its related signaling pathways (432). It was reported that co-treatment of breast cancer cells with phenethyl isothiocyanate and paclitaxel induced apoptosis, arrested the cell cycle, and inhibited cell growth (433, 434). Garcinol induced the death of the breast cancer cell lines MCF7, MDAMB231, and SKBR3 *via* triggering p53-dependent upregulation of Bax and downregulation of Bcl-xL (435). It also potentiated cisplatin sensitivity in HNSCC (436) and ovarian (437) cancer cells, as well as enhanced paclitaxel sensitivity in breast cancer cells (438) *via* inhibition of survivin, NF-κB/Twist-related protein 1 (Twist1), VEGF, caspase-3/calcium-independent phospholipase A2 (iPLA2), cyclin D1, Bcl-2, and PI3K/Akt signaling.

Hispidin (439), genistein (440, 441), guggulsterone (442), ginkgolide B (443), icariin (444), and zyflamend (445) potentiated the antineoplastic activity of gemcitabine in several cancer types, including pancreatic, osteosarcoma, and gallbladder cancers. Cisplatin in combination with tangeretin (446), galangin (447), and cepharanthine (448) decreased the proliferation and invasion of esophageal, lung, and ovarian cancer cells. Cotreatment of paclitaxel with icariside II (449) and caffeic acid (450), as well as combined treatment of 5-FU with oxymatrine (451), troxerutin (452), and calebin (453) increased the sensitivity of colon, lung, and melanoma cancer cells. Parthenolide (454) elevated oxaliplatin toxicity in A549 cells. The anticancer activity of doxorubicin was enhanced when combined with forbesione and isomorellin (455). Dioscin potentiated the effects of adriamycin in the K562 leukemia cell line (456).

In summary, phytochemicals have the potential to increase the sensitivity of various cancer cells and animal tumor models to several anticancer drugs. Phytochemicals augment chemoresistance through interfering with many processes, such as cell cycle arrest, DNA damage, angiogenesis, and variant signaling pathways, especially TLR/NF-κB/NLRP (**Table 5**).

**Table 5:** Phytochemicals in combination with anticancer drugs potentiate chemotherapy and immunotherapy: focusing on TLR/NF-κB/NLRP pathway.

Compound	Antineoplastic Agent	Types of study	Cell line(s)/cancer model(s)	Mechanisms of action	References
Curcumin	Paclitaxel	*In vitro* and *in vivo*	Xenograft model of human cervical cancer; Human cervical cancer (HeLa)	↓NF-κB; ↓tumor incidence; ↓tumor volume; ↓survival signals; ↓Akt; ↓MAPKs; ↓cell proliferation; ↓angiogenesis	(327)
	Paclitaxel	*In vitro*	Breast cancer cells (MDA-MB-231, MCF-7)	↑apoptosis; ↑necrosis	(329)
	Paclitaxel	*In vitro*	Breast cancer cells (MDA-MB-231, MCF-7)	↓NF-κB; ↓c-Ha-Ras; ↓Rho-A; ↓p53; ↓Bcl-XL; ↓Bcl-2	(328)
	Paclitaxel	*In vitro*	Cervical cancer cells (HeLa, CaSki)	↑apoptosis; ↑p53; ↑cleavage of caspase−3; ↓cell growth; ↓NF−κB−p53−caspase−3	(330)
	Paclitaxel & Cyclophosphamide	*In vitro* and *in vivo*	Breast cancer cells (MDA-MB-231, MCF-7); Swiss albino mice	↓NF-κB; ↓PKC; ↓HDAC; ↓telomerase	(331)
	Oxaliplatin	*In vitro*	Colorectal adenocarcinoma (LoVo, HT29, and DLD1)	↓Akt/NF-κB; ↓CXC-Chemokine/NF-κB; ↓NF-κB	(332)
	Cisplatin; Carboplatin; Oxaliplatin	*In vitro*	Human colorectal cancer cell (HT-29)	↓NF-κB; G2/M arrest; ↑apoptosis	(333)
	Dasatinib	*In vitro* and *in vivo*	Human colon cancer cell (SW-620, HCT-116, HT-29); Female min mice	↓NF-κB activity; ↓IGF-1R; ↓C-Src; ↓EGFRs; ↓cell growth	(334)
	Tamoxifen	*In vitro*	Breast cancer cells (MCF-7/LCC9 and MCF-7/LCC2)	↓NF-κB; ↓Akt/mTOR; ↓Src; ⟂ G2/M cell cycle arrest	(335)
	Retinoic acid	*In vitro*	Breast cancer cell lines (MDA-MB-231 and MD-MB-468)	↓FABP5; ↓PPARβ/δ; ↓VEGF-A; ↓PDK1	(336)
	Doxorubicin	*In vivo*	Xenograft 4T1 tumor-bearing mice	↓NF-κB	(337)
	Sorafenib	*In vitro* and *in vivo*	Hepatocellular carcinoma (Huh7); Athymic BALB/c nu/nu mice	↓NF-κB/p65; ↓MMP-9	(338)
Resveratrol	Cisplatin	*In vitro* and *in vivo*	Breast cancer cells (MDA-MB-231); Xenografts BALB/c mice	↓tumor growth; ↓TGF-β1; ↓Fibronectin	(483)
	Cisplatin	*In vitro*	Prostate cancer cell (PC3 and DU145)	↑Apoptosis; ↓COX-2; ↓NF-κB; ↑DUSP1	(339)
	Cisplatin	*In vitro*	Non-small cell lung cancer cell (NCI-H460)	↓Cell invasion; ↓cell migration; ↑apoptosis; ↓NF-κB; ↓Bcl-2; ↑caspase-3; ↑p53; ↑Bax; ↑p21; ⟂G0/G1 phases cell cycle	(340)
	5-fluorouracil (5-FU)	*In vitro*	Colorectal cancer cells (SW480R, HCT116)	↓IκBα kinase; ↓IκBα phosphorylation; ↓NF-κB	(341)
Apigenin	Paclitaxel	*In vitro*	Cervical cancer cell (HeLa)	↑Apoptosis; ↑ROS; ↓SOD activity; ↑cleavage of caspase-2	(343)
	Tamoxifen	*In vitro*	Breast cancer cells (MCF-7)	↑Apoptosis; ⟂G2/M phase cell cycle; ↑p53; ↓Cyclin B1	(344)
	Gemcitabine	*In vitro*	Pancreatic cancer cells (CD18, AsPC-1)	↓Cell proliferation; ⟂ G2/M and S phases cell cycle; ↑apoptosis; ↓pAkt	(346)
	Gemcitabine	*In vitro* and *in vivo*	Pancreatic cancer cells (MiaPaca-2, AsPC-1); Xenograft BALB/c nude mice	↓NF-κB; ↓tumor growth; ↓Akt; ↑apoptosis	(345)
	Doxorubicin	*In vitro*	Human prostate cancer cell (PC3)	↑Caspases; ↑Bax; ↑cyt c; ↓Bcl-XL; ↑p21; ↑p27; ↓Snail; ↓Twist; ↓MMPs; ↓pERK; ↑PTEN; ↓pPI3K; ↓pAkt	(347)
	Cisplatin	*In vitro*	Human prostate cancer cell (PC3)	↑Apoptosis; ↑Caspase-8; ↑Apaf-1; ↑p53; ↓Bcl-2; ↑p21; ⟂G2/M and S phases cell cycle	(348)
	Cisplatin	*In vitro* and *in vivo*	Human bladder cancer cells (UMUC2, T24); Swiss albino inbred mice	↓Tumor growth; ↑Mice survival	(353)
	Cisplatin	*In vitro* and *in vivo*	Oral squamous cell carcinoma (cell lines Tca-8113 and SCC-15); Xenograft mice models	↓NF-κB; ↑apoptosis; ↑caspase-8 and caspase-9; ↓xIAP	(352)
	Adriamycin	*In vitro* and *in vivo*	P388 leukemia cells; Xenograft mice models	↑NF-κB; ⟂S phase cell cycle; ↑Caspase-3; ↓Bcl-2; ↑Bax	(354)
	Gemcitabine	*In vitro*	Pancreatic cancer cells (PANC-1, BxPC-3); Hepatocellular carcinoma cell (Huh-7, HepG2)	⟂S phase cell cycle; ↑p53; ↓cyclin D1	(355)
EGCG	Sunitinib	*In vitro* and *in vivo*	Human lung carcinoma (H460 and H1975); Breast carcinoma cells (MCF-7); Xenograft mouse model	↓IRS/MAPK/p-S6K1; ↓PI3K/Akt; ↓MEK/ERK	(357)
	Cisplatin	*In vitro* and *in vivo*	Mice bearing Ehrlich ascites carcinoma; Cervical cancer cell (HeLa); Lung cancer cells (A549); Monocytic leukemia cell (THP-1)	↓NF-κB activation; ↓cyclin D1, ↓MMP-9, and VEGF	(356)
	Irinotecan	*In vitro*	Colorectal cancer cell (HCT116 and RKO)	↓Cell migration; ↓Cell invasion; ⟂S phase cell cycle; ⟂G2 phase cell cycle; ↓topoisomerase I; ↑DNA damage; ↑apoptosis; ↑autophagy	(358)
	Doxorubicin	*In vitro*	Bladder cancer cells (SW780 and T24)	↓NF-κB; ↓MDM2; ↑p53; ↑p21; ↑cleaved-PARP	(359)
	Gemcitabine	*In vitro* and *in vivo*	Pancreatic cancer cells (MIA PaCa-2 and Panc-1); C57BL/6J mice	↓Cell growth; ↓cell invasion; ↓cell migration; ↓Akt	(360)
	Cisplatin	*In vitro* and *in vivo*	Ovary cancer cell (OVCAR3, SKOV3); Xenograft mouse model	↑Cisplatin; ↑DNA-Pt adducts; ↑copper transporter 1	(361)
Naringin	Doxorubicin	*In vitro* and *in vivo*	Esophageal cancer stem cell (YM1); Xenograft mouse model	↓Tumor size; ↓systemic toxicity; ↓Cell viability; ↑apoptosis; ⟂S phase cell cycle	(362)
	Paclitaxel	*In vitro*	Prostate cancer cells (PC3, DU145, and LNCaP)	↓Cell survival; ↑apoptosis; ⟂ G1 phase cell cycle	(363)
Baicalin	Cisplatin	*In vitro*	Lung cancer cells (A549 and A549/DPP)	⟂S1 phase cell cycle; ↑apoptosis; ↑DNA damage; ↑Bax; ↓Bcl-2; ↓cyclin D1; ↓DNA repair	(364)
	Doxorubicin	*In vitro*	Breast cancer cells (MCF-7, MDA-MB-23)	↑ROS; ↑apoptosis	(365)
Arctigenin	Cisplatin	*In vitro*	Colorectal cancer cells (SW480 and SW620)	↑Autophagy; ↑apoptosis; ↑Cleaved caspase-3; ↑LC3-II; ↓LC3-I	(368)
	Cisplatin	*In vitro*	Non small lung cancer cell (H460)	↓Survivin; ⟂G1/G0 phase cell cycle; ↑apoptosis; ↑cleavage of caspase-3	(367)
	Cisplatin	*In vitro*	Hepatocellular cancer cells (HepG2); Human cervical cancer (HeLa)	↓STAT3; ↓pSTAT3; ↓Src; ↓JAK1; ↓JAK2; ↓ERK; ↓Akt	(366)
Morin		*In vitro*	Ovarian cancer cells (SK-OV-3, TOV-21G)	↓Cell viability; ↓cell proliferation; ↑apoptosis; ↑galectin-3	(369)
Chrysin	Doxorubicin	*In vitro*	Lung cancer cells (H157, H1975, A549, H460)	↓GSH; ↑cell death	(370)
Pterostilbene	Tamoxifen	*In vitro*	Breast cancer cells (ZR-751 and MCF7)	↓Cell viability; ↑apoptosis	(371)
Zerumbone	Cisplatin	*In vitro*	Human NSCLC cells (A549 and NCI-H460)	↑p53; ↑ROS	(372)
		*In vitro*	Prostate cancer cells (DU145 and PC3)	↓Cell growth; ↓JAK2; ⟂G0/G1 phase cell cycle; ↑apoptosis	(373)
Andrographolide	Doxorubicin	*In vitro*	Hepatocellular cancer cell (HepG2); Cervical cancer cell (HeLa); Breast cancer cell (MDA-MB-231); Colorectal cancer cell (HCT116)	↓JAK-STAT3 pathway; ↓pSTAT3; ↓JAK1/2; ↑Apoptosis	(374)
	Doxorubicin	*In vitro* and *in vivo*	Murine breast cancer cell (4T1); Xenograft BALB/c nude mice	↓Tumor growth; ↓HUVEC; ↓VEGFR2; ↓cell migration; ↓cell invasion	(375)
	Gemcitabine	*In vitro*	Pancreatic cancer cells (SW1990, Panc-1, AsPC-1, BxPC-3, and Capan-1)	↓STAT3; ↓Akt; ↑Apoptosis; ↑p21^Waf1^; ↑Bax; ↓Cyclin D1; ↓cyclin E; ↓survivin; ↓X-IAP; ↓Bcl-2	(376)
	Cisplatin	*In vitro* and *in vivo*	Human NSCLC cell (A549, LLC); C57BL/6 mice	↑Apoptosis; ↓LC3B-I; ↓LC3B-II; ↓Atg5	(377)
Carnosic acid	Cisplatin	*In vitro* and *in vivo*	Mouse Lewis lung cancer cell (LLC); C57BL/6 mice	↑IFN-γ; ↑FasL; ↑granzyme B; ↓MDSC; ↓iNOS2; ↓Arg-1; ↓MMP-9	(378)
	Tamoxifen	*In vitro* and *in vivo*	Breast cancer cells (MCF-7 and T47D); Mouse xenograft model	↑Apoptosis; ↑caspase-3; ↓Bcl-2; ↓Bcl-XL; ↑DcR1; ↑DcR2; ↑TRAIL; ↑Bax; ↑Bad	(379)
Triptolide	Cisplatin	*In vitro* and *in vivo*	Human gastric adenocarcinoma (AGS, SC-M1); SCID mouse xenograft model	↑Apoptosis	(380)
	Cisplatin	*In vitro*	Breast cancer cells (BT549, MDA-MB-231)	↑DNA breaks; ⟂S phase cell cycle; ↑DNA damage; ↓PARP1; ↓XRCC1; ↓RAD51	(382)
	Cisplatin	*In vitro*	Human bladder cancer cells (T24R2)	↑Caspase-9; ↑PARP; ↑cyt c; ↓pAkt; ↓pERK	(381)
	Paclitaxel	*In vitro* and *in vivo*	Human lung adenocarcinoma cell (A549); Xenografts balb/c-nude mice	↓Tumor growth; ↓tumor volume; ↓tumor size	(383)
	Hydroxycamptothecin	*In vitro*	Bladder cancer cell (EJ, UMUC3)	⟂G1 phase cell cycle; ↓CDK4; ↓CDK6; ↓Cyclin D1; ↓Akt	(385)
	Hydroxycamptothecin	*In vitro*	Pancreatic cancer cell (PANC-1)	↓NF-κB; ↑caspase-9, caspase-3	(384)
	Doxorubicin	*In vitro*	MDA-MB-231 and MCF7	↑Apoptosis; ↓ATM; ↑DDR; ↑DNA break	(387)
Ursolic acid	Gemcitabine	*In vitro* and *in vivo*	Human pancreatic cancer cells (Panc-28, MIA PaCa-2, AsPC-1); Orthotopic nude mouse model	↓NF-κB; ↓STAT3; ↓cell proliferation; ↑apoptosis; ↓angiogenic	(389)
	Oxaliplatin	*In vitro* and *in vivo*	Colorectal cancer cells (RKO, SW620, LoVo, SW480); Xenograft mouse model	↓NF-κB; ↓cell proliferation; ↑Apoptosis; ↓ERK1/2; ↓JNK; ↓Akt; ↓IKKα; ↓pMAPK; ↓PI3K/Akt;	(390)
	Cisplatin	*In vitro*	Human cervical cancer cells (C-33A, HeLa, ME-180, SiHa);	↓NF-κB p65; ↑apoptosis; ↓Cell growth; ↓Bcl-2	(391)
	Paclitaxel	*In vitro* and *in vivo*	Ovarian carcinoma cells (HEC-1A and OVCAR-3); Nude mouse xenograft model	↓PI3K/Akt/NF-κB; ↑apoptosis; ↑pJNK; ↓pAkt; ↑JNK	(388)
Oridonin	Cisplatin	*In vitro*	Human ovarian cancer cells (A2780, SKOV3)	↑Apoptosis; ↓MMP; ⟂G0/G1 phase cell cycle	(393)
	Gemcitabine	*In vitro*	Pancreatic cancer cell (PANC−1)	↓Bcl−2/Bax ratio; ↑Cyt c; ↑caspase−3; ↑caspase−9; ↑apoptosis; ⟂G1 phase cell cycle	(392)
	Doxorubicin	*In vitro*	Breast cancer cell (MDA-MB-231)	↑Apoptosis; ↓Bcl-2/Bax; ↓PARP; ↓caspase 3; ↓survivin; ↓blood vessel formation	(394)
	Cisplatin	*In vitro*	Lung cancer cell (A549/DDP); Xenograft BABL/c mice	↑Tumor inhibition	(395)
Ginsenoside Rg3	Paclitaxel	*In vitro* and *in vivo*	Breast cancer cell (BT-549, MDA-MB-231, MDA-MB-453); Xenograft BALB/c nu/nu mice	↓NF-κB	(396)
	Cisplatin	*In vitro* and *in vivo*	Human NSCLC cell lines (H1299, SPC-A1, and A549); Xenograft tumor mice model	↓NF-κB	(398)
	Cisplatin	*In vitro*	Human NSCLC cell line (A549)	↓NF-κB p65; ↓PD-L1; ↓Akt	(397)
	Gefitinib	*In vitro*	Human NSCLC cell line (H1299, A549)	↓Cell migration; ↑Bax; ↑cleaved-caspase-3; ↓Bcl-2	(399)
	5-FU	*In vitro*	Esophageal squamous cell carcinoma (TE-1, ECA-109); Lung cancer cells (H460)	↑DNA damage; ↓DNA repair; ↓DNA replication; ↓CHEK1 degradation; ↓autophagy	(400)
	Docetaxel	*In vitro*	Colon cancer cells (HCT116, SW620)	↓NF-κB; ↑Bax; ↑caspase-3; ↑Caspase-9	(401)
	Docetaxel	*In vitro*	Prostate cancer cells (DU145, LNCaP, PC-3)	↓NF-κB; ↑apoptosis; ⟂G0/G1 phase cell cycle	(402)
Lycopene	Cisplatin	*In vitro*	Cervical cancer cell (HeLa)	↓NF-κB; ↓cell viability; ↑Bax; ↓Bcl-2; ↑Nrf-2	(403)
Berberine	Doxorubicin	*In vitro*	Lung cancer cell (NCI-H1975, NCI-H460)	↓STAT3; ↓cell proliferation; ↑Apoptosis	(404)
	Doxorubicin	*In vitro*	Breast cancer cell (MCF-7)	↓Nanog; ↓miRNA-21	(405)
	Doxorubicin	*In vitro*	Breast cancer cell (4T1)	↓HMGB1-TLR4 axis	(406)
	Cisplatin	*In vitro*	Ovarian cancer cell (A2780)	↓miR-93/PTEN/Akt; ↑Apoptosis; ⟂G0/G1 phase cell cycle; ↓miR-93	(407)
	Cisplatin	*In vitro*	Breast cancer cell (MCF-7)	↓Cell growth; ↑DNA breaks; ↑Apoptosis; ↑Capase-3; ↑Cleaved capspase-3; ↑Caspase-9; ↓Bcl-2	(408)
	Irinotecan	*In vitro*	Colon cancer cells (HCT116)	↓NF-κB; ↓c-IAP1, c-IAP2, survivin and Bcl-XL	(409)
Piperlongumine	Oxaliplatin	*In vitro* and *in vivo*	Gastric cancer cell (BMS-582949, SP600125); Nude mice xenograft model	↑ROS; ↓TrxR1; ↑DNA damage; ↑p38; ↑JNK	(414)
	Oxaliplatin	*In vitro* and *in vivo*	Colon cancer cells (HCT-116, LoVo); Xenograft mouse model	↑Oxidative stress; ↑ROS; ↑Apoptosis	(413)
	Cisplatin	*In vitro* and *in vivo*	HNSCC (AMC-HN3 and AMC-HN9); BALB/c athymic nude mice	↑ROS; ↑JNK; ↑PARP; ⟂Sub-G1 phase cell cycle	(410)
	gemcitabine	*In vitro* and *in vivo*	Pancreatic cells (PCNA and Ki-67); Xenograft mouse model	↓NF-κB	(411)
	Doxorubicin	*In vitro* and *in vivo*	Breast cancer cells (MDA-MB-231, MDA-MB-453); Mice models	↑Apoptosis; ↓JAK2/STAT3; ↓cell growth; ↑apoptosis	(412)
Harmine	Paclitaxel	*In vitro*	Gastric cancer cells (SGC-7901 and MKN-45)	↓Cell migration; ↓cell invasion; ↓MMP-9; ↓COX-2	(416)
	Paclitaxel	*In vitro*	Gastric cancer cell (SGC-7901)	↓Bcl-2; ↑Bax; ↓PCNA; ↓COX-2	(417)
	Gemcitabine	*In vitro*	Pancreatic cancer cells (BxPC-3, CFPAC-1, PANC-1, SW-1990)	↑Apoptosis; ↓cell proliferation; ↑cleavage of PARP; ↑caspase-3; ↓Akt/mTOR	(418)
Matrine	Irinotecan	*In vitro*	Colorectal cancer cell (HT29)	↑Apoptosis; ↑topoisomerase I; ↑Bax; ↑caspase-3	(419)
	Cisplatin	*In vitro* and *in vivo*	Mouse cervical cancer cell (U14); Kunming mice	↑TSLC1; ↓tumor growth	(422)
Sophoridine	Cisplatin	*In vitro* and *in vivo*	Lung cancer cells (NCIH446, NCI-H1299, NCI-H460, A549); Xenograft model in BALB/c mice	↑p53; ↑Hippo signaling	(423)
Sulforaphane	Everolimus	*In vitro*	Bladder cancer cells (TCCSUP, RT112, UMUC3)	↓Cell growth; ↓cell proliferation; ↑p19; ↑p27; ↓phosphorylation of CDK1; ↓CDK1; ↓cyclin B; ⟂S phase cell cycle	(427)
	Oxaliplatin	*In vitro*	Colorectal cancer cells (Caco-2)	↓Cell proliferation; ↓ATP; ↑DNA cleavage; ↑caspase-3	(424)
	Gefitinib	*In vitro*	Lung adenocarcinoma cell (PC9)	↓Cell proliferation; ↓SHH; ↓SMO; ↓GLI1	(426)
	Paclitaxel	*In vitro*	Breast cancer cell lines (MDA-MB-231 and MCF-7)	↓NF−κB	(425)
	Salinomycin	*In vitro* and *in vivo*	Colorectal cancer cells (Caco-2 and CX-1); Xenografted nude mice	↓PI3K/Akt; ↑p53; ↑apoptosis; ↓Bcl-2; ↑Bax; ↑Bax/Bcl-2 ratio; ↑PARP cleavage	(428)
Shikonin	Gemcitabine	*In vitro* and *in vivo*	Pancreatic cancer cell (BxPC-3, PANC-1, AsPC-1); Xenograft mouse model	↓NF-κB; ↓tumor growth; ↓Cell proliferation; ↓micro vessel density; ↑apoptosis;	(429)
	Gefitinib	*In vitro* and *in vivo*	Human NSCLC cells (HCC827, H1299, A549, H1975); Nude mice	↑PKM2; ↓cell proliferation; ⟂G0/G1 phase cell cycle; ↑apoptosis; ↓PKM2/STAT3/cyclin D1	(430)
	Paclitaxel	*In vitro*	Esophageal cancer cells (KYSE270, KYSE150)	↑Apoptosis; ↓cell growth; ↓cell mitotic; ↓Bcl-2; ↑p53	(431)
	4-hydroxytamoxifen	*In vitro* and *in vivo*	Breast cancer cell lines (MCF−7 and MDA−MB−435S); BALB/c mice model	↑Apoptosis; ↓mitochondrial membrane potential; ↑ROS; ↓PI3K/AKT/caspase 9	(432)
Phenethyl isothiocyanate	Paclitaxel	*In vitro*	Breast cancer cells (MCF7, MDA-MB-231)	↓Cell growth; ↑Apoptosis; ⟂G2/M phase cell cycle	(433)
	Paclitaxel	*In vitro*	Breast cancer cells (MCF7, MDA-MB-231)	↑Apoptosis; ↑acetylation of alpha-tubulin; ↓Cdk1; ↓Bcl-2; ↑Bax; ↑cleavage of PARP	(434)
Garcinol	Cisplatin	*In vitro*	Ovarian cancer cells OVCAR-3	↓NF-κB; ↓PI3K/Akt phosphorylation; ↑Bax; ↓p-PI3K; ↓pAkt proteins; ⟂S phase cell cycle; ↑apoptosis	(437)
	Paclitaxel	*In vitro* and *in vivo*	Breast cancer cell (4T1); Balb/c mice metastasis model	↓NF-κB/Twist1; ↓caspase-3/iPLA2; ⟂G2/M phase arrest	(438)
Hispidin	Gemcitabine	*In vitro*	Pancreatic cancer cells (BxPC-3 and AsPC-1)	↓NF-ĸB; ↓cell proliferation; ↓Bcl-2; ↑tumor suppressor p53; ↑cleaved caspase-3; ↑cleaved PARP	(439)
Genistein	Gemcitabine	*In vitro*	Osteosarcoma cells (MG-63 and U2OS)	↓Akt/NF-κB	(440)
Genistein	Gemcitabine	*In vitro* and *in vivo*	–	↓NF-κB; ↓Akt	(441)
Ginkgolide B	Gemcitabine	*In vitro*	Pancreatic cancer cell (BxPC-3 and CAPAN1)	↓PAFR/NF-кB	(443)
Icariin	Gemcitabine	*In vitro* and *in vivo*	Human gallbladder carcinoma cell (GBC-SD and SGC-996); BALB/c (nu/nu) mice	↓NF-κB; ⟂G0/G1 phase arrest; ↓Bcl-2; ↓Bcl-XL	(444)
Zyflamend	Gemcitabine	*In vitro* and *in vivo*	Pancreatic cancer cell (AsPC-1, BxPC-3, MIA PaCa-2, PANC-1); Orthotopic mouse model	↓NF-κB	(445)
Tangeretin	Cisplatin	*In vitro*	Human lung cancer cell (A549, A2780)	↓NF-κB; ↓PI3K/Akt; ↓apoptosis; ↓p-Akt; ↓phospho-GSK-3β; ↓phospho-BAD; ⟂G2-M phase arrest	(446)
Galangin	Cisplatin	*In vitro* and *in vivo*	Mice xenograft model	↓NF-κB; ↓STAT3; ↓Bcl-2/Bax; ↓p-STAT3/p65; ↓Bcl-2	(447)
Cepharanthine	Cisplatin	*In vitro* and *in vivo*	Human ESCC cell line (Eca109); BALB/c nude mice	↑TNFR1-JNK; ↓Bcl-2	(448)
Icariside II	Paclitaxel	*In vitro*	Human melanoma cell (A375)	↑Apoptosis; ↑cleaved caspase-3; ↓IL-8; ↓VEGF; ↓TLR4	(449)
Caffeic acid	Paclitaxel	*In vitro*	Human lung cancer cell (A549 and H1299)	↓NF-κB	(450)
Oxymatrine	5-FU	*In vitro*	Colon cancer cell (HCT-8/5-FU)	↓NF-κB; ↓vimentin	(451)
Troxerutin	5-FU	*In vitro* and *in vivo*	Human gastric cancer (SGC7901); Mice xenograft model	↓STAT3/NF-κB; ↓Bcl-2	(452)
Calebin a	5-FU	*In vitro*	Colorectal cancer cell (HCT116)	↓p65-NF-κB	(453)
Parthenolide	Oxaliplatin	*In vitro*	Human lung cancer cell (A549)	↓NF-κB; ↑apoptosis; ↓COX-2; ↓PGE2	(454)
Forbesione and isomorellin	Doxorubicin	*In vitro*	Human cholangiocarcinoma cells (KKU-100, KKU-M139, KKU-M156)	↓NF-κB; ↑Bax/Bcl-2; ↑caspase-9; ↓survivin	(455)
Dioscin	Adriamycin	*In vitro*	Leukemia K562 cell	↓NF-κB ↓MDR1	(456)

## Nanoformulations of Phytochemicals Against Chemoresistance and Immunotherapy Resistance

Mutations and long-term chemotherapy lead to the development of chemoresistance, prompting the need for progressively increasing dosages of anticancer drugs. Consequently, these higher concentrations of chemotherapeutic agents are toxic to noncancerous cells (457). Nanoparticles are increasingly used due to their improved bioavailability, protection of drug molecules, high specificity for cancer cells, and decreased clearance. Combining the versatile capabilities of nanoparticles with the aforementioned benefits of phytochemicals created the concept of phytonanomedicine, which revolutionized cancer therapy (458). Phytonanomedicine utilizes the valuable properties of phytochemicals merged with the nano-size, high surface area, optical activity, and surface reactivity of nanoparticles to achieve active or passive tissue-specific drug delivery. The application of phytonanocompounds may reduce the toxicity and side effects of chemotherapeutic agents, while increasing their efficacy, thereby combating chemoresistance (459).

Flavones are the most prominent natural phytochemical that regulates the functions of Bax, Bid, and Bak proteins. Additional mechanisms by which phytochemicals combat chemoresistance are altering the expression of selected genes during mitosis or meiosis, regulating the expression of mutated genes like p21 and p53, and interfering with DNA repair mechanisms. Liposomes, polymeric nanoparticles, polymeric micelles, nanodispersion, and dendrimers, among others, are efficient nanocarriers that are often used (460).

Quercetin-loaded mesoporous silica decorated with chondroitin sulfate potentiated the delivery of paclitaxel, overcoming MDR in breast cancer cells. Such co-administration successfully targeted CD44 receptor-mediated targeting with a low half-maximal inhibitory concentration (IC_50_) value by increasing cell cycle arrest in the G2/M phase and destroying microtubules. Quercetin also decreased paclitaxel efflux by downregulating the expression of P-gp (349). In another study, Zafar et al. (461) used phosphorylated chitosan nanoparticles loaded with α-lipoic acid to overcome chemotherapy resistance. The nanodelivery system was able to cross the cell barrier and expose the MDA-MB-231 cells to a high load of the therapeutic agent. Similar nanoparticles were also used for loading quercetin and paclitaxel simultaneously (462). The efficacy of curcumin and temozolomide co-delivery to chemoresistant cells was highlighted by Bagherian et al. (458). In this study, the curcumin nanomicelles demonstrated high cytotoxic effects against U87 cells by attenuating apoptotic and autophagic mediators. Zafar et al. (461) also used a phytochemical, thymoquinone, to prevent chemoresistance to docetaxel (e.g., endosomes escape). The neoplastic agent was loaded in chitosan nanoparticles and exhibited cytotoxicity against MCF-7 and MDA-MAB-231 cancer cell lines. Recently, a functionalized dendrimer has also been employed to co-deliver siRNA and curcumin to target HeLa cancer cells. Such co-administration increased the cytotoxicity against cancer cells by reducing Bcl-2 and improving apoptosis (463). Baicalein nanoparticles targeted folate and hyaluronic acid to display anticancer effects on paclitaxel-resistant lung cancer cells by decreasing tumor growth and cell viability (464). The nanosuspension of another flavonoid, chrysin, showed anticancer effects against HepG2 cells by blocking cell growth (465). The phytosome of luteolin, another flavonoid, exhibited anticancer activity against MDA-MB-231 cells by reducing the expression of Nrf-2/HO-1 and decreasing cell viability (466). Poly-lactic acid (PLA)-polyethylene glycol (PEG) nanoformulation of luteolin also demonstrated anticancer effects against TU212 HNSCC cells, H292 lung cancer cells, and a xenograft mouse model of head and neck cancer (467).

Nanostructured lipid carriers were also used for dual drug loading of imatinib and curcumin to target the CD20 receptor of lymphoma cells. This co-delivery system displayed promising responses in resistant tumor cells (468). By regulating oxidative stress and apoptosis, phyto-nanocomposites diffuse into the organelles of cancer cells and overcome chemoresistance through multiple signaling pathways, including TLR/NF-κB/NLRP, ERK/MAPK/JNK, stress-activated protein kinase/JNK, TRAIL, PI3K/Akt, and p53/caspase mediated apoptotic pathways (469).

Poly(lactic-co-glycolic acid nanoparticles of 4-methyl-7-hydroxy coumarin (a synthetic coumarin) and dendrosomal nanoformulation of farnesiferol c (a coumarin) demonstrated anticancer effects by decreasing cell proliferation in gastric cancer cells and by modulating the Bax/Bcl-2 ratio (470). Other phytochemicals, such as flavonolignans PEG nanoliposomes, silibinin, and glycyrrhizic acid, demonstrated anticancer effects by decreasing cell viability in HepG2 cells (471).

A nanoformulation of honokiol, a lignan, showed anticancer effects in lung cancer cell lines by inducing cell cycle arrest in the G0/G1 phase (459). Silver nanoparticles of plumbagin, a naphthoquinone, induced apoptosis in the human skin cancer cells HaCaT and A431, producing free radicals and increasing pyruvate kinase activity (472). Liposomal phytochemicals may possess anticancer effects in intravenous usage. The liposomal carrier significantly increased plasma levels of curcumin in a dose-dependent manner in cancer patients. In one clinical study, liposomal curcumin showed acceptable plasma concentration with a significant but temporary decrease in tumor markers such as prostate-specific antigen and carcinoembryonic antigen, in patients with metastatic tumors. Additionally, liposomal curcumin inhibited sphingosine kinase (473). Lipocurcumin is known to be a safe drug that significantly suppresses cancer markers *via* apoptosis and cell cycle arrest. The curcumin nanoparticle suppressed STAT3/NF-κB, thereby inducing apoptosis. Accordingly, the phase Ib/IIa clinical trial showed no drug-related severe toxicity while maintaining an inhibitory effect on cancer resistance. Such nanoparticles could target cancer-specific mediators in solid tumors (473).

The hyaluronic acid-modified PEGylated liposomes of doxorubicin-stigmasterol were used against chemoresistant breast cancer (474). Gold nanoparticles have shown promising potential in both cancer chemotherapy and immunotherapy (475). Resveratrol gold nanoparticles improved antitumor activity through mitochondrial accumulation and apoptosis both *in vitro* and *in vivo*. Gold nanoresveratrol also elevated the expression of caspase-8 and Bax, while reducing pro-caspase-3 and pro-caspase-9. Ginseng-derived nanoparticles are another phytonanocompound that induced M2 to M1 macrophage polarization through TLR4 and MyD88 signaling. It also produced ROS and increased apoptosis of melanoma cells (476).

Various nanoparticles may represent a novel class of nano-targeted systems in cancer immunotherapy and chemotherapy (**Table 6**).

**Table 6:** Phytonanocompounds against chemoresistance, by targeting TLR/NF-κB/NLRP pathway and interconnected mediators.

Compound	Nanoparticle type	Type of study	Cell line(s) cancer model(s)	Mechanisms of action	References
Curcumin	NLCs; liposome	*In vitro*	Cervical cancer cell (HeLa); Glioblastoma cells (U87 MG); Prostate cancer cell lines (LNCaP & C4-2B)	↓Bcl-2; ↑apoptosis; ↓PSA, CEA, CA 19-9; ↑sphingosine kinase inhibitory activity	(463, 477)
Quercetin	Different nanoparticles	*In vitro* and *in vivo*	Brest cancer cell line; Human prostate cancer (PC-3); Nude male BALB/c mice	↑cd44 activity; ↑apoptosis; ↑G2M phase arrest; ↓P-gp expression	(349)
Coumarins	Dendrosome	*In vitro*	Gastric adenocarcinoma (AGS)	↑Apoptosis; ↑DNA fragmentation; ↑caspase-3	(470)
Silibinins	Nanoliposome	*In vitro*	Hepatocellular carcinoma cell (HepG2)	↓Cell viability	(471)
Resveratrol	Nanoparticles based on poly(epsilon-aprolactone) and poly(D,L-lactic-co-glycolic acid)-poly(ethylene glycol); Gold NPs	*In vitro* and *in vivo*	Prostate cancer cells (PC-3, LNCaP, DU-145)	↓Cell growth & proliferation; ↑ROS; ↑caspase-3; caspase-8; ↑Apoptosis; ↑Bax; ↓pro-caspase-3; pro-caspase-9	(478)
Honokiol	Nanomicellar	*In vitro* and *in vivo*	Lewis lung cancer LL/2 cell lines	↑Cell cycle arrest at G0/G1 phase	(459)
Plumbagin	Different nanoparticles	*In vitro*	Epidermoid carcinoma cell (HacaT, A431)	↑Free radicals; ↑pyruvate kinase activity	(472)
Biacalein	Different nanoparticles	*In vitro*	Human lung cancer cell (A549)	↓Cell viability	(465)
Luteolin	Different nanoparticles	*In vitro*	Breast cancer cells (MDA-MB-231); Human lung cancer cell (H292)	↓Nrf-2 expression; ↑Akt	(466)
Ginseng	Different nanoparticles	*In vivo* and *in vitro*	Melanoma cells	↑TLR4; ↑ROS; ↑M1 macrophages	(476).
Taxan alkaloids	Liposome & polymeric micelle formulation	*In vivo*	Gastric cancer; Prostate cancer cell lines (PC3, DU145, and LNCaP)	↓Cell survival; ↑apoptosis; ⟂G1 phase cell cycle	(479)
Taxan alkaloids	Liposome	*In vitro*	Colon cancer cells (HCT116, SW620); Prostate cancer cells (DU145, LNCaP, PC-3)	↓P-gp	(401)
Vinca alkaloids	Liposome	*In vitro*	Colorectal cancer cell (HCT116 and RKO)	↑DNA damage; ↑cell cycle arrest	(358)

## Conclusion, Current Limitations, and Future Perspectives

Agrowing number of reports have highlighted the difficulty of chemoresistance when administering chemotherapy and immunotherapy. Several complex pathophysiological mechanisms behind chemoresistance have been discovered. Of those mechanisms, TLR/NF-κB/NLRP has been identified as a crucial aspect in the development of chemoresistance. The TLR/NF-κB/NLRP pathway is interconnected with several inflammatory, oxidative stress, and apoptotic mediators. Thus, there is an urgent need to develop novel multi-targeted agents to overcome drug resistance and restore the sensitivity of current chemotherapeutic drugs. Plant secondary metabolites (e.g., polyphenols, alkaloids, terpenes/terpenoids, and sulfur compounds) are potential multi-targeted anticancer agents that may combat chemoresistant dysregulated mediators. We have also reviewed the potential of phytochemicals in the modulation of the tumor microenvironment (13, 33, 480). Despite the effectiveness of plant secondary metabolites in regulating chemoresistance-associated pathways, their low solubility, poor bioavailability, instability, and low selectivity limit clinical efficacy and therapeutic uses in cancer treatment. The applicability of nanotechnology has greatly improved bioavailability, cellular uptake, efficacy, and specificity of anticancer phytochemicals, thereby overcome those pharmacokinetic limitations (6, 13, 457, 481). In line with this, liposomes, polymeric nanoparticles, micelles, nanodispersion, nanostructured lipid carriers, and dendrimers of plant secondary metabolites have played pivotal roles in reducing chemoresistance (482).

**Figure 8:**
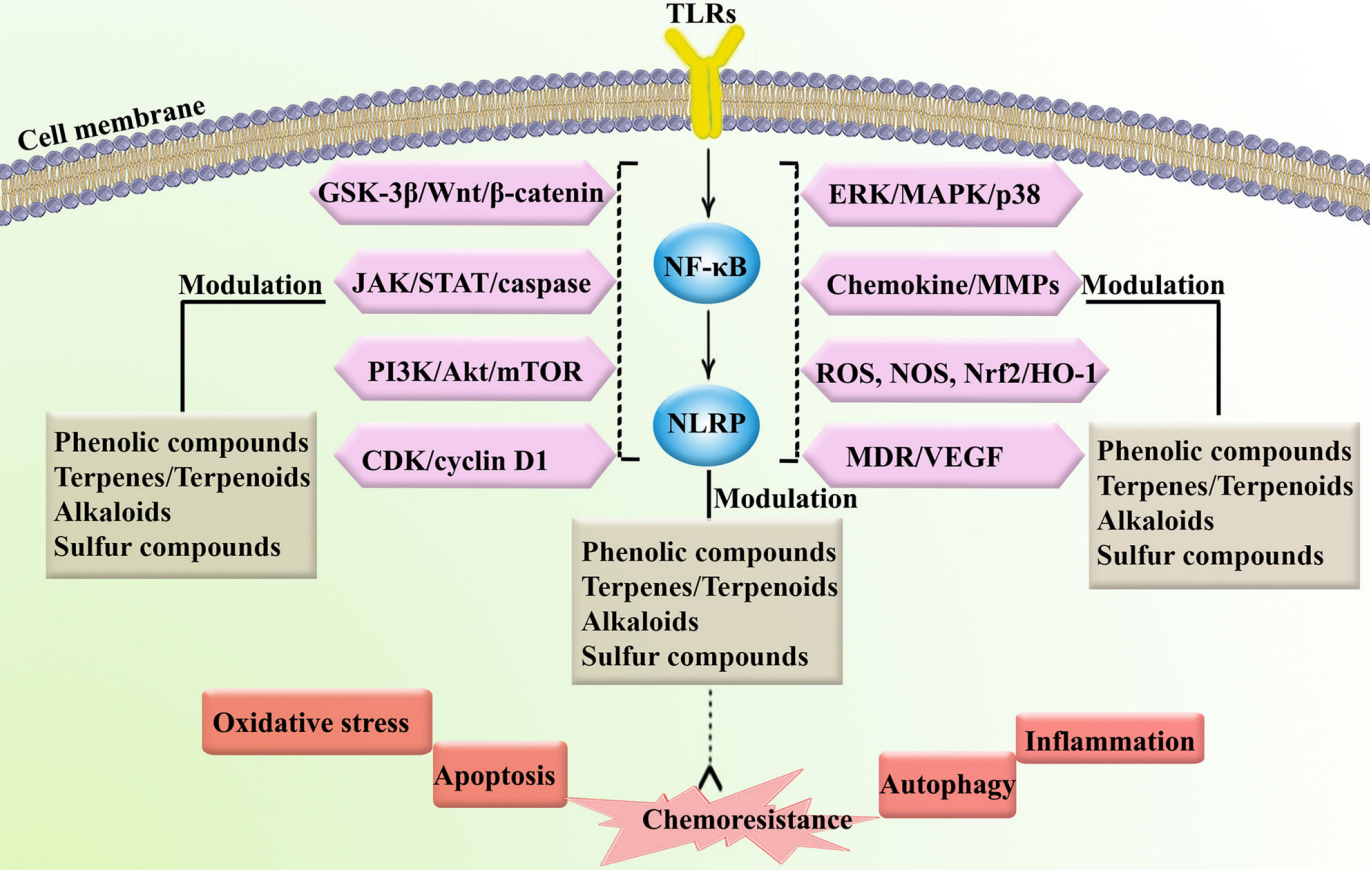
Targeting chemoresistance-related signaling pathways and interconnected mediators by phytochemicals. CDK, cyclin-dependent kinase; ERK, extracellular-regulated kinase; GSK-3, glycogen synthase kinase-3; HO-1, heme oxygenase 1; JAK, Janus kinase; NOS, nitric oxide synthase; MAPK, mitogen-activated protein kinase; MDR, multidrug resistance; MMP; matrix-metalloproteinase; mTOR, mammalian target of rapamycin; NLRP, nod-like receptor pyrin domain-containing; NOS, nitric oxide synthase; Nrf-2, nuclear factor-erythroid factor 2-related factor 2; PI3K, phosphoinositide 3-kinases; ROS, reactive oxygen species; STAT, signal transducer and activator of transcription; VEGF, vascular endothelial growth factor.

In this systematic and comprehensive review, the critical roles of phytochemicals have been highlighted in the modulation of TLR/NF-κB/NLRP to combat chemoresistance. The need to develop novel delivery systems of plant secondary metabolites and targeted therapy is also highlighted. Further research should be performed involving additional dysregulated mechanisms in chemoresistance which may reshape therapeutic approaches to utilize plant-derived secondary metabolites (**Figure 8**). Additional studies should consist of extensive *in vitro* and *in vivo* experimentation to further unveil emergent chemoresistance signaling pathways, as well as well-controlled clinical trials. Such reports may reform current therapeutic strategies, allowing treatment methods to obtain higher potency/efficacy with fewer side effects and less chemoresistance by using phytochemicals that target the TLR/NF-κB/NLRP signaling pathway.

## Data Availability Statement

The original contributions presented in this study are included in the article/supplementary material. Further inquiries can be directed to the corresponding author.

## Author Contributions

Conceptualization, AB and SF. Literature search and collection: SF, SM, AY, and FN. Writing-original draft: SF, SM, AY, and FN. Writing-review and editing, SF, CW, and AB. Supervision: AB. Project administration: AB. All authors contributed to the article and approved the submitted version.

## Conflict of Interest

The authors declare that the research was conducted in the absence of any commercial or financial relationships that could be construed as a potential conflict of interest.

## Publisher’s Note

All claims expressed in this article are solely those of the authors and do not necessarily represent those of their affiliated organizations, or those of the publisher, the editors and the reviewers. Any product that may be evaluated in this article, or claim that may be made by its manufacturer, is not guaranteed or endorsed by the publisher.
